# Low cost and massively parallel force spectroscopy with fluid loading on a chip

**DOI:** 10.1038/s41467-022-34212-w

**Published:** 2022-11-10

**Authors:** Ehsan Akbari, Melika Shahhosseini, Ariel Robbins, Michael G. Poirier, Jonathan W. Song, Carlos E. Castro

**Affiliations:** 1 Department of Mechanical and Aerospace Engineering, The Ohio State University, Columbus, OH 43210 USA; 2 Department of Physics, The Ohio State University, Columbus, OH 43210 USA; 3 Biophysics Graduate Program, The Ohio State University, Columbus, OH 43210 USA; 4 Department of Chemistry and Biochemistry, The Ohio State University, Columbus, OH 43210 USA; 5 Comprehensive Cancer Center, The Ohio State University, Columbus, OH 43210 USA

**Keywords:** Single-molecule biophysics, Characterization and analytical techniques, Microfluidics

## Abstract

Current approaches for single molecule force spectroscopy are typically constrained by low throughput and high instrumentation cost. Herein, a low-cost, high throughput technique is demonstrated using microfluidics for multiplexed mechanical manipulation of up to ~4000 individual molecules via molecular fluid loading on-a-chip (FLO-Chip). The FLO-Chip consists of serially connected microchannels with varying width, allowing for simultaneous testing at multiple loading rates. Molecular force measurements are demonstrated by dissociating Biotin-Streptavidin and Digoxigenin-AntiDigoxigenin interactions along with unzipping of double stranded DNA of varying sequence under different dynamic loading rates and solution conditions. Rupture force results under varying loading rates and solution conditions are in good agreement with prior studies, verifying a versatile approach for single molecule biophysics and molecular mechanobiology. FLO-Chip enables straightforward, rapid, low-cost, and portable mechanical testing of single molecules that can be implemented on a wide range of microscopes to broaden access and may enable new applications of molecular force spectroscopy.

## Abstract (web summary)

Single molecule force spectroscopy methods are often low throughput and have high instrument cost. Here the authors report FLO-Chip, a low-cost, high throughput technique using microfluidics for multiplexed mechanical manipulation of many individual molecules via molecular fluid loading on-a-chip.

## Introduction

Advances in molecular biophysics have been propelled by several force spectroscopy techniques such as magnetic tweezers, atomic force microscopy, and optical tweezers^1–6^. However, these techniques require the use of complex, specialized, and often cost-prohibitive instrumentation for most laboratories^7,8^ that are often low-throughput, usually testing one or a few interactions at a time. Recent developments in centrifugal force microscopy^9^, acoustic bead manipulation^10^, wide-range magnetic field^11^, and nanophotonic bead trapping^12^ have helped reduce the total cost per force spectroscopy experiment by enabling multiplexed manipulation and loading of molecular constructs. Despite these attempts towards reducing the cost of force spectroscopy while increasing the throughput, further improvement efforts towards cost-efficiency and simplified instrumentation can help make single-molecule force spectroscopy available to most laboratories. Notably, hydrodynamic force spectroscopy^13–18^ is a simple, lost cost technique that has enabled multiplexed application of mechanical load in molecular constructs. While this approach sacrifices some advantages of other methods, such as rapid force modulation of acoustic^10^ or optical manipulation^12^, and the calibration-free force estimation offered by centrifugal force microscopy^9^, it provides a simple, low-cost technique that can be carried out in a wide range of laboratories. However, the current state-of-the-art hydrodynamic force spectroscopy techniques are limited to a single, straight channel enabling testing for a single level of mechanical load or dynamic loading rate. Since full characterization of molecular interactions requires application of a wide range of mechanical forces, it is of great advantage to develop a cost-efficient, high-throughput, and easily portable force spectroscopy approach with standard sample preparation and instrumentation that can enable testing of multiple levels of mechanical force simultaneously.

This study presents a low-cost, high throughput technique using microfluidics for massively parallel application of molecular fluid loading on-a-chip (FLO-Chip) to test various molecular interactions under a wide range of mechanical loads (~0.1–100 pN) and loading rates (10^−2^−10^2^ pNs^−1^). Utilizing fluid flow to apply mechanical tension on molecular interactions is easily implementable without the need for specialized instrumentation^13,15,19^. Moreover, our approach utilizes polydimethylsiloxane (PDMS)-based soft lithography to enable production of minimal cost flow cells (~3$ per chip, see Supplementary Table 1) bonded on chemically functionalized coverslips^20^. The implemented flow cells consist of multiple microchannels with different widths that are serially connected. Since the flow velocity is a function of the channel geometry, having channels with different widths enables execution of multiple irreversible single-molecule bond rupture tests under varying loading conditions (e.g., loading rates) on one chip resulting in significantly enhanced experimental throughput (up to ~4000 measurements per chip within ~2 hours of total experiment time). In addition, these tests can be evaluated using standard imaging equipment that is widely available to most biology, biophysics, biomaterials, and bioengineering laboratories, thereby eliminating the need for specialized force spectroscopy instrumentation and broadly increasing access to single-molecule force studies.

For verification, we study the extension of double-stranded DNA (dsDNA) tethers under controlled tension and reproduce the previously reported worm-like chain behavior^21,22^. Furthermore, we examine the dissociation of biotin-streptavidin and digoxigenin (DIG)-AntiDIG (digoxigenin antibody) along with unzipping of double-stranded DNA with different stem length and sequences under various dynamic loading rates and solution conditions. These experiments yield most probable rupture forces and binding interaction free energy landscape parameters that agree well with prior results using more complex force spectroscopy techniques. Therefore, our approach enables a high-throughput single-molecule force spectroscopy on-a-chip with low-cost and simple preparation procedure that can easily be integrated into a wide range of laboratories for rapid physical characterization of molecular complexes.

## Results

### FLO-Chip design and force calibration

**Fig. 1:**
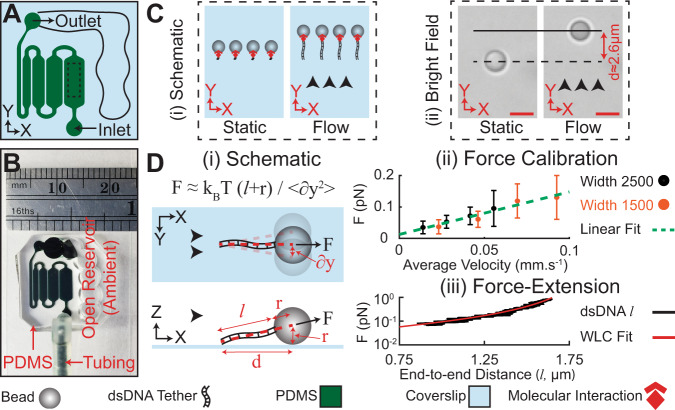
Hydrodynamic force spectroscopy on-a-chip. **A**, **B** Schematic and representative photograph of the microfluidic platform used in fluid loading on a chip (FLO-Chip). **C** (i) Schematic, and (ii) Differential Interference Contrast (DIC) image of beads anchored to the coverslip via a single dsDNA tether. Application of flow causes ~2.6μm displacement of the bead center as the result of the drag force applied on the bead. Scale bars, 2 μm. **D** (i) Schematic of the direction of in-plane and off-plane stretching force (F) applied on a tether bead under flow. Application of F leads to transverse fluctuation of the tethered bead. (ii) Calibration of F with respect to the inlet perfusion rate (Q) in the 1500 μm and 2500 μm wide microchannel. Transverse fluctuation of *n* = 33 beads in the 2500 µm wide channel and *n* = 23 beads in the 1500 µm wide channel were monitored over three independent experiments under different flow rates. Error bars represent standard deviation. The calibration line (red dash line) indicated a linear relationship between drag force and perfusion rate with slope of ~1.4 ± 0.2 pN s mm^−1^. Error bars represent standard deviation. (iii) Force-extension examination of single tethers under varying F. The tether length (l) was well described according to the worm-like chain model (solid red line, persistence length = 56 ± 8 nm, contour length = 1.8 ± 0.1 µm). Black arrowheads denote flow direction. Scale bars are 2 μm. Source data are provided as a Source Data file.

The throughput of force spectroscopy is often limited by one-at-a-time loading as in optical tweezers or atomic force microscopy (AFM) measurements. Methodologies such as magnetic tweezers or centrifugal force microscopy allow multiplexed application of mechanical loading^8^, but they remain limited to what interactions can be tracked in a single field of view because all interactions in the assay (including ones that are not being monitored) experience the same loads. Here we overcame this limitation in multiplexing through a microfluidic chip design that consists of multiple, serially connected microchannels with varying width, allowing for loading some samples to rupture while limiting loads in other channels. This enabled execution of multiple flow rupture tests per chip resulting in significantly enhanced throughput. Figure 1 illustrates the design approach in FLO-Chip with multiple microchannels of varying width (500 µm, 1000 µm, 1500 µm, 2000µm, and 2500 µm) and fixed height (120 µm) that were serially connected. The local drag force scales with flow velocity, and the flow velocity is inversely proportional to the width of the channel for a constant channel height, which is the case here. Hence, for a given volumetric flow rate, the narrowest channel exhibits the largest force followed by the channels of increasing width that experience 1/2, 1/3, 1/4, and 1/5 the force and loading rate, respectively. This design was chosen to enable multiple sets of force spectroscopy measurements per chip, either under similar (i.e., repeats) or distinct loading conditions, resulting in significantly enhanced throughput (Fig. 1A, B).

To enable utilization of fluid flow to mechanically manipulate binding interactions, we sandwiched the molecular construct of interest between a microbead (2.2 µm average diameter) on one side and a 5745 bp double-stranded DNA (dsDNA) tether anchored to a glass coverslip on the other side. The dsDNA tether was anchored to a glass surface by biotin-streptavidin binding. Subjecting the anchored samples to fluid flow causes a drag force on the microbeads that stretches the dsDNA tethers and translates the drag force to the molecule or interaction of interest (Fig. 1C). The net resultant lift force due to combination of wall effect and shear gradient in Poiseuille flow near the coverslip surface provides a force in the upward direction towards the center of the channel. This force counteracts the downward vertical component of the force on the tether and would tend to minimize non-specific interactions between the bead and the coverslip surface (Fig. 1Di, bottom)^23,24^.

The input parameter in FLO-Chip is the perfusion rate, Q (i.e. volumetric flow rate), controlled using a syringe pump. However, to use FLO-Chip for single-molecule force spectroscopy, the level of drag force that is applied on tethered microbeads (F) when subjected to perfusion needed to be calibrated. Therefore, as a first step, we estimated the level of drag force (F) applied on the beads with respect to the perfusion rate (Q). To this end, we subjected the tethered beads to a range of perfusion rates and monitored the mean-square displacement of the bead center in the transverse direction by video monitoring (Fig. 1Di; Supplementary Fig. S1A). The level of F with respect to Q was calibrated based on the equipartition theorem according to equation:

1 $${{{{{\rm{F}}}}}}=\frac{{{{{{{\rm{k}}}}}}}_{{{{{{\rm{B}}}}}}}{{{{{\rm{T}}}}}}\,({{{{{\rm{l}}}}}}+{{{{{\rm{r}}}}}})}{{ < {{{{{\rm{\delta }}}}}}{{{{{\rm{y}}}}}}}^{2} > }$$

where ${{{{{{\rm{k}}}}}}}_{{{{{{\rm{B}}}}}}}$ is the Boltzmann constant, ${{{{{\rm{T}}}}}}$ is the absolute temperature, ${{{{{\rm{l}}}}}}$ is the end-to-end length of the DNA tether, r is the bead radius, and ${ < {{{{{\rm{\delta }}}}}}{{{{{\rm{y}}}}}}}^{2} >$ is the variance of bead center displacement in the direction transverse to the flow (Fig. 1D i, ii; Supplementary Fig. S2)^25,26^. In addition, by monitoring the in-plane displacement of the bead along the direction of the flow, the distance between the center of the bead and the base of the dsDNA tether near the coverslip surface $({{{{{\rm{l}}}}}}+{{{{{\rm{r}}}}}})$ can be estimated based on the following equation (Fig. 1Di; Supplementary Fig. S1A):

2 $${({{{{{\rm{l}}}}}}+{{{{{\rm{r}}}}}})}^{2}\, \approx \,{{{{{{\rm{d}}}}}}}^{2}+{{{{{{\rm{r}}}}}}}^{2}$$

Estimation in Eq. 2 is based on negligible vertical displacement of the beads subjected to flow compared to in-plane displacement of the bead in the direction of the flow. This was confirmed by monitoring traces of tethered beads next to stuck beads when subjected to different flow rates. No significant off-plane displacement of tethered beads was detected compared to stuck beads (Supplementary Fig. S1B).

This approach has been previously implemented to estimate the tether stretching force in hydrodynamic^15^ and magnetic tweezer^27^ based force spectroscopy. We performed calibration in two of the microchannels (1500 µm and 2500 µm in width) by monitoring displacement of up to 30 beads per microchannel when subjected to four different perfusion flow rates (Supplementary Fig. S3). Since the sampling frequency must be greater than the limiting Lorentzian sampling frequency to enable accurate monitoring of bead lateral fluctuations^27^, we performed calibration at <~1 pN forces. As anticipated, F increased linearly with respect to Q in each channel. The slope of the linear fit was used as the calibration coefficient to determine F for any given Q. Moreover, the slope extracted from each linear fit decreased proportionally with the microchannel width which was anticipated as the average flow velocity decreases proportionally to the width of each channel for any given flow rate (Supplementary Fig. S3). Therefore, we calibrated the values for F estimated based on Eq. 1 with respect to the average flow velocity in each of the two calibrated channels (Fig. 1Diii; Supplementary Fig. S3). The linear fit revealed a slope of 1.4 ± 0.2 pN.s.mm^−1^ that was used to estimate F in each FLO-Chip microchannel. It is worth noting that the performed calibration is only valid for beads of the same diameter (2.2 ± 0.2 µm), tether length (~1.9 µm), and microchannel height (120 µm). The FLO-Chip must be recalibrated if any of these factors change when performing single-molecule force spectroscopy experiments.

### Force-extension behavior of dsDNA

To verify the estimated F with respect to average perfusion velocity, we used FLO-Chip to study the extension behavior of the dsDNA tether under force. We subjected beads that were anchored to the coverslip via a single tether to a force ramp and monitored the increase in ${{{{{\rm{l}}}}}}$ upon the increasing F (Fig. 1D i, iii) as estimated based on Eq. 2. The average force-extension curve was well described by the worm-like chain (WLC) model^21,22^ for dsDNA (persistence length = 56 ± 8 nm, contour length = 1.8 ± 0.1 µm, total of nine beads analyzed). The extracted average persistence length and contour length were in good agreement with values reported for dsDNA persistence length^28^ and the anticipated contour length value for the 5745 bp tether (~1.95 µm assuming 0.34 nm length per bp for dsDNA)^29^. These results verify the validity of using the force versus volumetric flow rate calibration to estimate F, and demonstrate the capability to study the force-extension behavior of biopolymers.

### Rupture forces of protein-small molecule interactions

A key goal of the FLO-Chip system is to enable testing of forces required to rupture molecular interactions. The FLO-Chip assay requires at least two molecular interactions: one to anchor one of the dsDNA tether ends to the coverslip surface, and another to attach the other end of the tether to the microbead. In principle, since the tether stretching force is applied on both interactions simultaneously, FLO-Chip can be used to examine the rupture force required to dissociate either interaction. However, this would require synthesis of a new tether for every interaction of interest. To enable a versatile setup that can incorporate various molecular interactions for rupture force measurements, we used DNA base-pairing to bind the interaction of interest onto the tether. In addition, the assay requires stable binding to the surface. Hence, we prepared a 5745 bp dsDNA tether with biotin at one end, for surface attachment, and a 30nt ssDNA overhang at the other end, for attaching the interaction of interest. Based on previous studies, we estimated biotin-streptavidin would withstand force up to ~40 pN, and a 30 bp DNA duplex (in a shearing configuration) would withstand forces up to 42-55 pN at the loading rates of interest (~0.1–10 pNs^−1^)^30,31^. Hence, this setup could be used to probe interactions with rupture forces up to ~40 pN over a range of loading rates ~0.1–10 pNs^−1^.

**Fig. 2:**
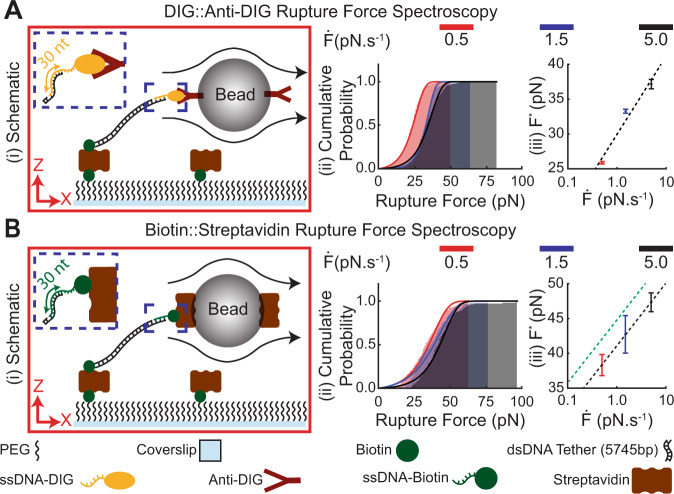
Hydrodynamic force spectroscopy of the Biotin-Streptavidin and digoxigenin (DIG)-AntiDIG binding interaction. **A**(i) Schematic of the setup used to examine the dissociation force of DIG and Anti-DIG binding interaction. The tether included a DIG end that enables binding to beads functionalized with Anti-DIG and a biotin end that enables binding of the tether to the streptavidin on the coverslip surface. (ii) Cumulative probability histogram of the recorded rupture forces under three different loading rates and (iii) the most probable rupture force (F^*^) with respect to applied loading rate ($\dot{{{{{{\rm{F}}}}}}}$) for DIG-AntiDIG binding interaction. N=3 most probable rupture forces were estimated based on random selection without replication of the recorded rupture force data under each loading rate. The estimated F^*^ values are presented as mean values ±SD. **B**(i) Schematic of the setup used to execute hydrodynamic force spectroscopy on biotin-streptavidin binding interaction. The tether included two biotin ends that enable binding of the tether to beads functionalized with streptavidin and to the streptavidin on the coverslip surface. (ii) Cumulative probability histogram of the recorded rupture forces under three different loading rates and (iii) F^*^ with respect to the applied $\dot{{{{{{\rm{F}}}}}}}$ for biotin-streptavidin binding interaction. *N* = 3 most probable rupture forces were estimated based on random selection without replication of the recorded rupture force data under each loading rate. The estimated F^*^ values are presented as mean values ±SD. For both binding interactions tested, the recorded rupture forces increased with increase in the applied $\dot{{{{{{\rm{F}}}}}}}$ under semilogarithmic scale. The linear fit performed (black dash lines) indicated $\triangle {{{{{\rm{X}}}}}}$ of 0.76 ± 0.05 nm and 0.95 ± 0.02 nm along with ${{{{{{\rm{k}}}}}}}_{{{{{{\rm{off}}}}}}}$ of 4.0 ± 1.4 × 10^−4^ s^−1^ and 4.7 ± 2.6 × 10^−6^ s^−1^ for DIG-AntiDIG and biotin-streptavidin binding interactions, respectively. Furthermore, the extracted energy landscape coefficients for biotin-streptavidin were used to project the most probable rupture force with respect to loading rate for a single biotin-streptavidin binding interaction (green dash line). Source data are provided as a Source Data file.

As proof of concept, we studied well-characterized interactions, starting with digoxigenin (Dig) binding with an anti-Digoxigenin antibody (AntiDig), which we expected to rupture at forces of ~20 pN within loading rate range of ~0.1–10 pNs^−110^^32^^,^. To this end, we annealed a ssDNA with DIG with complementary sequence to the free end of the tether to enable binding to microbeads coated with AntiDIG (Fig. 2Ai). We tested the strength of DIG-AntiDIG binding interactions under three different loading rates ($\dot{{{\mbox{F}}}}$=0.5, 1.5, and 5.0 pNs^−1^) (Fig. 2A ii,iii). To control the loading rate, we subjected the tethered beads to a single ramp of linearly increasing inlet flow rate to apply the desired mechanical loading rate based on the performed calibration in Fig. 1Diii. We observed a statistical distribution of rupture events, which shifted to higher forces with increasing loading rate, as illustrated in Fig. 2A,ii in terms of cumulative distribution functions. The most probable rupture force (F^*^) under each loading rate was determined from the cumulative distribution of rupture forces based on the Evans and Ritchie rupture model^33^ (Supplementary Fig. S4, see the Supplementary Material for further details). We measured rupture forces for DIG-AntiDIG of F^*^=25.9 ± 0.2 pN, 33.2 ± 0.3 pN and 37.2 ± 0.7 pN under loading rates of 0.5 pNs^−1^, 1.5 pNs^−1^, and 5.0 pNs^−1^, respectively, which agrees well with previous studies using atomic force microscopy (~30 pN under ~100 pN/s loading rate)^32^ and acoustic force spectroscopy (~26 pN under ~2 pN/s loading rate)^10^. To test whether the observed rupture forces for DIG-AntiDIG is dependent on the channel width, we subjected the DIG-AntiDIG binding interaction to the same dynamic loading rate in the 500 µm wide, the 1000 µm wide, and the 2000 µm wide channels and monitored the rupture force. The cumulative probability of DIG-AntiDIG rupture under dynamic force in three different channels was not dependent on the channel width (Supplementary Fig. S5).

According to Evans and Ritchie^33^, the most probable rupture force F* can be defined as:

3 $${{{{{{\rm{F}}}}}}}^{*}=\frac{{{{{{{\rm{k}}}}}}}_{{{{{{\rm{B}}}}}}}{{{{{\rm{T}}}}}}}{\triangle {{{{{\rm{X}}}}}}}{{{{{\rm{ln}}}}}}\frac{\dot{{{{{{\rm{F}}}}}}}\triangle {{{{{\rm{X}}}}}}}{{{{{{{\rm{k}}}}}}}_{{{{{{\rm{B}}}}}}}{{{{{\rm{T}}}}}}.{{{{{{\rm{k}}}}}}}_{{{{{{\rm{off}}}}}}}}$$

where ${{{{{{\rm{k}}}}}}}_{{{{{{\rm{off}}}}}}}$ is the dissociation rate at zero force, $\triangle {{{{{\rm{X}}}}}}$ is the distance to the transition state and $\dot{{{{{{\rm{F}}}}}}}$ is the applied loading rate. The extracted F* as a function of loading rate (Fig. 2A,iii) was fit to the Evans and Ritchie model (Eq. 3) to determine k_off_ = 4.0 ± 1.4 × 10^−4^ s^−1^ and *ΔX* = 0.76 ± 0.05 nm for the DIG-AntiDIG binding interaction, which are in the range of previously reported values^10,32^.

Next, we tested Biotin-Streptavidin, another well-characterized interaction^34^. In this case, we annealed single-stranded DNA oligos modified with Biotin to the ssDNA end of the tether along with using beads coated with Streptavidin (Fig. 2B,i). Similar to DIG-AntiDIG, the dissociation force for biotin-streptavidin binding is lower than the level of rupture force for DNA unbinding in shear^34,35^. Therefore, we anticipated the rupture to occur due to dissociation of the biotin-streptavidin interaction near the bead or the coverslip surface. It is worth noting that both tether ends could possibly bind to the glass coverslip preventing anchoring of the streptavidin-coated beads. As implemented previously by Yang et al.^9^, limiting the number of available streptavidin covering the coverslip surface can enable tethers that are only bound to the coverslip surface on one end. However, significant reduction in experimental throughput (~400 data points per chip) occurred when tethers with Biotin on both ends were utilized. This yield could likely be increased by varying the ratio between PEG and Biotin-PEG on the glass coverslip, but the throughput was reasonable for our analysis. We tested biotin-streptavidin binding under three different loading rates and observed a similar shift to larger rupture forces with increasing loading rate (Fig. 2B ii,iii). Similar to DIG-AntiDIG, the F^*^ values for biotin-streptavidin under each loading rate were extracted from the cumulative distributions of rupture forces (see the Supplementary Material for further details). Since three identical biotin-streptavidin interactions are connected serially under this configuration, bond dissociation under mechanical load ideally is three times more likely to occur compared to a single biotin-streptavidin binding interaction, which results in slightly weakened attachment. According to Evans^36^, the most probable rupture force for N serially connected binding interactions can theoretically be described as:

4 $${{{{{{\rm{F}}}}}}}^{*}=\frac{{{{{{{\rm{k}}}}}}}_{{{{{{\rm{B}}}}}}}{{{{{\rm{T}}}}}}}{\triangle {{{{{\rm{X}}}}}}}{{{{{\rm{ln}}}}}}\frac{\dot{{{{{{\rm{F}}}}}}}\triangle {{{{{\rm{X}}}}}}}{{{{{{{\rm{N}}}}}}.{{{{{\rm{k}}}}}}}_{{{{{{\rm{B}}}}}}}{{{{{\rm{T}}}}}}.{{{{{{\rm{k}}}}}}}_{{{{{{\rm{off}}}}}}}}$$

Patel et al. previously showed Eq. 4 accurately describes the rupture force of two iminobiotin-streptavidin binding interactions that were serially connected compared to the corresponding rupture force for a single iminobiotin-streptavidin interaction in AFM measurements^37^. Therefore, the F* values we obtained for biotin-streptavidin with respect to loading rate using FLO-Chip were fit with the modified Evans model (Eq. 4) revealing k_off_ = 4.7 ± 2.6 × 10^−6^ s^−1^ and ΔX=0.95 ± 0.02 nm. These values were used to determine the rupture force a single binding interaction according to Eq. 3. The projected values for F* with respect to loading rate along with the extracted values for k_off_ were in the range of previously reported values for biotin-streptavidin binding, while the extracted value of ΔX = 1.0 nm is slightly higher compared to previously reported values (~0.5 nm)^34,38^. The observed discrepancy in ΔX may be due the effect of the elastic worm-like chain DNA tether polymer connecting the biotin-streptavidin binding interactions as described by Evans^36^. Equation 4 is based on the main assumption that identical N serially connected interactions experience the same load history resulting in N-fold faster rate of construct rupture compared to a single interaction. Figure 2B schematic, however, depicts a single biotin-streptavidin interaction near the bead surface connected to two biotin-streptavidin interactions near the coverslip surface via a long elastic dsDNA tether. Therefore, when pulled far from equilibrium under a dynamic load, the force history experienced by the interaction near the bead surface may differ from the force history experienced by the interactions near the coverslip surface resulting in altered bond energy landscape for the entire complex compared to a single binding interaction^39^. Moreover, the discrepancy in the observed biotin-streptavidin distance to transition state using FLO-Chip can be attributed to the effect of force-loading geometry on characterization of biotin-streptavidin binding interaction. The tetravalency of streptavidin with respect to biotin can enable multiple directionalities of force applied on biotin with respect to the anchored streptavidin subunit^40^. Recent study using atomic force microscopy reported a significant increase in distance to transition state when biotin binds streptavidin subunit that are not the same as the anchored subunit^40^. Further analysis using streptavidin variants that enable control of force directionality can help better characterization of biotin-streptavidin binding interaction in FLO-Chip.

### Rupture forces for unzipping of dsDNA

**Fig. 3:**
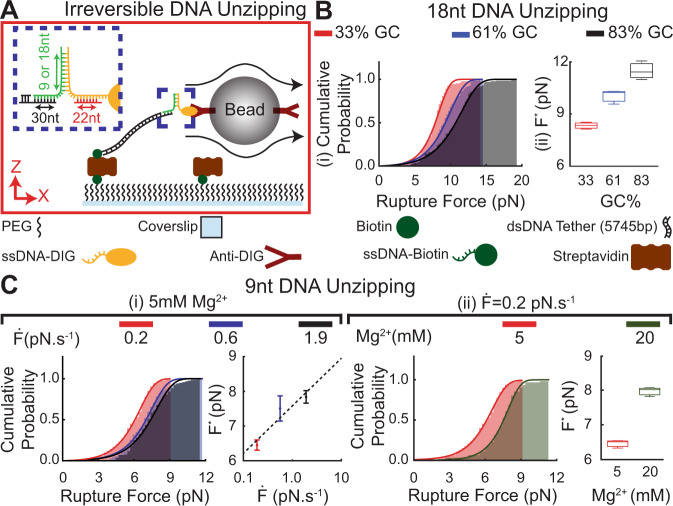
Assessment of double stranded DNA unzipping using hydrodynamic force spectroscopy. **A** Schematic of the setup used for mechanical unzipping of dsDNA. The DNA tether features a Biotin end that enables anchoring of the tether to the coverslip surface and a single-stranded end that binds to a short oligo (green oligo). A fraction of the green oligo enables binding to the tether, and another fraction enables binding to another short oligo conjugated to DIG (orange oligo). Similarly, a fraction of the orange oligo enables binding to the green oligo, and another fraction enables binding to a third short oligo (red oligo). **B** Cumulative probability histogram of DNA unzipping force for 18nt dsDNA with 33%, 58%, and 83% GC content. Increase in GC content caused the rupture events to occur at higher forces. *N* = 3 most probable rupture forces (F^*^) were estimated based on random selection without replication of the recorded rupture force data for each GC content. The estimated F^*^ values are presented as median (center), 25–75 percentile (box) and minima-maxima (whisker). **C** Cumulative probability histogram of DNA unzipping events for 9nt dsDNA with 78% GC content under (i) varying loading rates, and (ii) varying Mg^2+^ concentration in the buffer. *N* = 3 most probable rupture forces were estimated based on random selection without replication of the recorded rupture force data under each loading rate. The estimated F^*^ values under different loading rates are presented as mean values ±SD. The estimated F^*^ values under different Mg^2+^ concentrations are presented as median (center), 25–75 percentile (box), and minima-maxima (whisker). Rupture events occurred at higher forces with increase in loading rate and Mg^2+^ concentration. The most probable rupturing force increased linearly with loading rate under semilogarithmic scale. Performing a linear fit indicated $\triangle {{{{{\rm{X}}}}}}$ of 6.2 ± 0.4 nm along with ${{{{{{\rm{k}}}}}}}_{{{{{{\rm{off}}}}}}}$ of 4.0 ± 2.0 × 10^−6 ^s^−1^ for the 9nt DNA unzipping interaction. Source data are provided as a Source Data file.

Binding between two complementary ssDNA strands has recently been implemented to develop a variety of molecular mechanical probes^41^. We used FLO-Chip to quantify the rupture force required to unzip these dsDNA mechanical probe interactions. To this end, we annealed a ssDNA oligonucleotide with the sequence of interest to the single-stranded end of the tether that contained the complementary sequence followed by an additional sequence to attach to a complementary strand modified with DIG (Fig. 3A). This yielded an assay with the desired DNA binding interaction (in an unzipping configuration) sandwiched between the tether and a bead coated with Anti-DIG (Fig. 3A). We also annealed a third oligo (red in Fig. 3A inset) that was complementary to a fraction of the DIG-conjugated oligo to fortify the distance between the dsDNA unzipping interaction and the AntiDIG coated bead. First, we examined the role of GC content in regulating the force required to unzip an 18 bp long dsDNA duplex of varying sequence. To enable direct comparison of the FLO-Chip force spectroscopy data with previous studies, we used the same 18 bp dsDNA duplex sequence with 83% GC content that was previously used as a cellular tension sensor^42^. We subjected the interactions to a 0.19 pNs^−1^ loading rate in 5 mM Mg^2+^ and observed an increase in rupture force with increase in GC content (Fig. 3Bi), which is consistent with prior studies^43,44^. Fitting the Evans and Ritchie model^33^ (see Supplementary Material for further details) to each cumulative probability rupture force distribution revealed an increase in the most probable rupture force with increasing GC content (F^*^ = 8.3 ± 0.6 pN, 10.2 ± 0.6 pN and 11.6 ± 0.3 pN for 33%, 61%, and 83% GC content, respectively) (Fig. 3Bii). Previous studies revealed a force of 12–14 pN for the 18 bp DNA duplex with 83% GC content^42,45^, which is in close agreement with our value of F^*^ = 11.6 pN. It is worth noting that the reported 12–14 pN rupture force reported for the 18 bp dsDNA duplex with 83% GC content is the rupture force under which 50% of the interactions rupture in <2 seconds under constant mechanical load, whereas our studies allow for direct quantification of the most probable rupture force at a given loading rate.

Next, we used FLO-Chip to probe the rupture force for a 9 bp dsDNA duplex sequence that was also previously used in the design of hairpin-based cellular tension probes^44,46^. We subjected the interaction (78% GC) to various loading rates and observed increasing loading rates resulted in higher unzipping forces (Fig. 3Ci). The results revealed most probable rupture forces for the 9nt DNA unzipping interaction of F^*^=6.5 ± 0.2 pN, 7.5 ± 0.4 pN, and 7.8 ± 0.2 pN under loading rates of 0.2 pNs^−1^, 0.6 pNs^−1^, and 1.9 pNs^−1^, respectively. Previous measurements using biomembrane probe force spectroscopy on unzipping of a DNA hairpin with a stem sequence identical to the 9nt unzipping interaction tested at 500 pNs^−1^ revealed F^*^ of ~9.8 ± 3.4 pN^46^, which is in close agreement with the projected value of ~11.3 pN estimated based on the loading rate dependence determined here (Fig. 3Ci). The slight discrepancy in rupture force is likely due to the effect of the stem loop in the DNA hairpin. Moreover, the reported 9.8 pN force for unzipping the 9 bp dsDNA hairpin is the value at which the interaction exhibits 50% probability of unzipping, which is theoretically lower than the most probable rupture force reported here. Fitting the most probable rupture force versus loading rate results to the Evans and Ritchie model (Eq. 3) revealed a dissociation rate at zero force of k_off_=4.0 ± 2.0 × 10^−6^ s^−1^ and a distance to transition state of ΔX = 6.2 ± 0.4 nm for the 9nt DNA unzipping. These values were in the range of coefficients reported for DNA hairpin unzipping interactions with similar stem length and GC content examined using optical trap force spectroscopy^43^.

An important parameter influencing the stability of DNA binding interactions is the cation concentration. We tested the effects of increasing Mg^2+^ concentration, which is known to increase DNA melting temperatures^47^. Increasing the Mg^2+^ concentration from 5 mM to 20 mM under 0.2 pN.s^−1^ loading rate caused the F^*^ to increase from 6.5 ± 0.2 pN to 8.0 ± 0.3 pN. These results are consistent with the expected increase in stability and demonstrate the capacity to use the FLO-Chip assay to test interactions under a variety of solution conditions.

### Massively parallel hydrodynamic force spectroscopy under multiplexed fluid loading rates

**Fig. 4:**
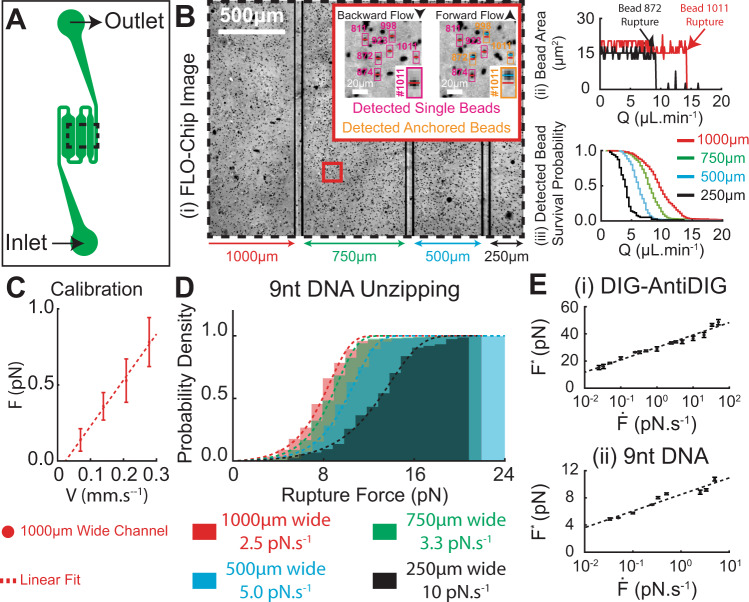
Massively parallel dynamic force spectroscopy under multiplexed dynamic loading rates. **A** Schematic of FLO-Chip outline depicting serially connected microchannels with varying width. **B** (i) Representative bright-field image of the FLO-Chip microchannels (250 µm, 500 µm, 750 µm, and 1000 µm in width) coated with beads tethered to the coverslip using DIG-AntiDIG binding interaction. The zoomed-in images of coated FLO-Chip depicting selection of beads that are tethered to the surface via a single tether. The difference between the detected bead center position under backward flow (red solid line) and forward flow (cyan solid line) was used to select tethered beads. (ii) The rupture flow rate for each selected bead was determined by monitoring the bead area when the beads ware subjected to linearly increasing flow rate. The flow rate at which each bead was undetected was marked as the corresponding rupture flow rate. (iii) Probability of the selected beads to remain bound under increasing flow rate. Bead rupture was more probable under smaller flow rates in narrower channels. **C** Calibration of bead drag force (F) with respect to the inlet average velocity (V) in the 1000 μm wide microchannel. *N* = 30 beads were monitored over three independent experiments. The calibration line (red dash line) indicated a linear relationship between drag force and average perfusion velocity with slope of ~3.0 ± 0.2 pN s mm^−1^. **D** Dynamic force spectroscopy of 9nt DNA unzipping with 78% GC content under four different loading rates tested simultaneously on the same chip. **E** Dynamic force spectroscopy of (i) DIG-AntiDIG dissociation and (ii) 9nt DNA unzipping tested under a wide range of loading rates (10^−2^−10^2^ pN.s^−1^ and 10^−2^−10^1^ pN.s^−1^, respectively). *N* = 3 most probable rupture forces (F^*^) were estimated based on random selection without replication of the recorded rupture force data under each loading rate. The estimated F^*^ values are presented as mean values ±SD. Source data are provided as a Source Data file.

Multiplexed testing of different loading rates on the same chip can significantly enhance the throughput of rupture force characterization under a wide range of dynamic mechanical loads. To enable multiplexing of fluid loading rates in a single experiment, we designed a different version of the FLO-Chip that allows for channels of varying width (250 µm, 500 µm, 750 µm, and 1000 µm) and fixed height (60 µm) to be imaged simultaneously in one field of view (Fig. 4A, Bi). To automate identification of beads tethered to the surface via a single DNA tether, the bead positions were monitored when subjected to two opposite fluid flows that cause stretching of the DNA tether in each direction. The beads that exhibited significant movement (greater than ~2 µm) parallel to the flow direction when subjected to each fluid flow were selected for force spectroscopy analysis (Fig. 4 Bi). Furthermore, detection of a rupture event was also automated by monitoring the bead pixel area when subjected to a linearly increasing flow rate. Upon rupture, the bead immediately flows away, and the flow rate when the pixel area drops to zero is marked as the corresponding rupture flow rate (Fig. 4 Bii). This automation allows for rapid characterization of the full probability distribution of rupture forces simultaneously in all microchannels, each subjected to a distinct loading rate. Since the average velocity and stretching force increase proportionally with decreasing channel width, bead rupture occurs first in the narrowest channel followed by beads in the channels with increasing widths (Fig. 4 Biii). This automated bead selection and rupture flow rate estimation allowed for highly efficient analysis of the time-lapse images (~5 minutes total analysis time for ~4000 beads using MATLAB).

Similar to the previous FLO-Chip design, we estimated the level of drag force (F) applied on the beads with respect to the perfusion average velocity (V). We performed calibration in the widest microchannel (1000 µm in width) by monitoring displacement of ~30 beads per microchannel when subjected to four different perfusion flow rates. As anticipated, F increased linearly with respect to V (Fig. 4C). The linear fit revealed a slope of 3.0 ± 0.2 pN s mm^−1^, which was used to estimate F in each FLO-Chip microchannel.

Using this chip, we repeated dynamic force spectroscopy measurements on DIG-AntiDIG dissociation (four chips tested for four sets of loading rates in the 10^−2^−10^2^ pNs^−1^ range, ~4000 beads analyzed per chip) and 9nt DNA unzipping (three chips tested for three sets of loading rates in the 10^−2^−10^1^ pNs^−1^ range, ~2000 beads analyzed per chip). Representative cumulative probability rupture force distributions for 9nt DNA unzipping extracted from a single experiment on one chip illustrates the ability to test four different loading rates simultaneously (Fig. 4D). The resulting F* versus loading rate for DIG-AntiDIG (Fig. 4 Ei) was fit to the Evans and Ritchie model^33^ (Eq. 3) to determine k_off_ = 7.9 ± 3.2 × 10^−5 ^s^−1^ and ΔX = 1.1 ± 0.1 nm for the DIG-AntiDIG binding interaction. Similarly, for the 9nt DNA unzipping interaction, the F* versus loading rate (Fig. 4 Eii) was fit to the Evans and Ritchie model^33^ (Eq. 3) to determine k_off_ = 2.2 ± 0.7 × 10^−4 ^s^−1^ and ΔX = 4.1 ± 0.3 nm for the 9nt DNA unzipping interaction. The estimated values of k_off_ and ΔX for DIG-AntiDIG dissociation were in close agreement with the corresponding values estimated in Fig. 2Aiii. However, the estimate value of k_off_ for 9nt DNA unzipping interaction was 2-orders of magnitude larger from Fig. 4E ii compared to the corresponding value extracted from Fig. 3Ci. Since the range of loading rates and number of analyzed beads were larger in Fig. 4E ii compared to Fig. 3C i, we report k_off_ = 2.2 ± 0.7 × 10^−4 ^s^−1^ and ΔX = 4.1 ± 0.3 nm for the 9nt DNA unzipping interaction.

### FLOChip enables investigation of the effect of mechanical strain on toehold-mediated strand displacement reaction

Toehold-mediated strand displacement is a widely implemented technique in programming dynamic rearrangement of DNA-based nanoconstructs^48–50^. In this approach, one strand in a dsDNA duplex can be displaced through the addition of an “invader” strand. To achieve the displacement, an ssDNA extension is added to one of the two strands, referred to as the “substrate,” in the dsDNA duplex. The other strand, which gets displaced, is referred to as the “incumbent.” The invader strand is designed to fully complement the substrate strand, such that it can first bind the ssDNA extension (i.e., the toehold) and then displace the incumbent through branch migration^48^. Therefore, the rate of the toehold-mediated strand displacement reaction is regulated by both the rate of toehold binding and branch migration. While DNA hybridization to a mechanically strained substrate has been investigated^51,52^, the impact of mechanical force on toehold-mediated strand displacement remains largely unknown. FLOChip can enable massively parallel monitoring molecular dissociation events at a single-molecule level under multiple levels of mechanical force providing a tremendous advantage for studying the force-sensitivity of toehold-mediated strand displacement.

**Fig. 5:**
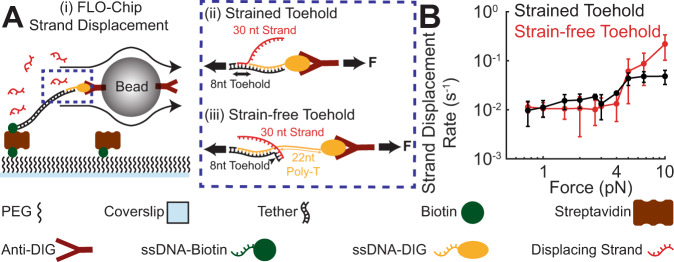
Investigating the impact of toehold strain on strand displacement reaction rate using FLOChip. **A** i) Schematic demonstrating implementation of FLOChip to investigate strand displacement reaction rate under force. ii) Schematic of the configuration that enables application of force to both the toehold and the branch migration domain. iii) Schematic of the configuration that enables the application of force to only the branch migration domain while the toehold domain remains strain-free. **B** Impact of strain on toehold-mediated strand displacement reaction rate under 0.75–10 pN force range under Strained Toehold (both the toehold and the branch migration domains are under strain, black data set) alongside Strain-free Toehold (only the branch migration domain is under strain while the toehold domain remains strain-free, red data set). *N* = 3 strand displacement rates were estimated based on random selection without replication of the recorded dissociation kinetics data under each mechanical load. The estimated F^*^ values are presented as mean values ±SD. Source data are provided as a Source Data file.

To test the effect of mechanical force on toehold-mediated strand displacement in FLOChip (Fig. 5Aii, strained toehold), we annealed a Dig-modified oligo complementary to the ssDNA end of the DNA tether leaving an 8nt toehold on the tether side of the Dig-modifed oligo (Fig. 5Aiii, strain-free toehold). In this configuration, the fluid flow subjects both the toehold and branch migration domains to force. We further studied a second construct consisting of a Dig-modified oligo complementary to the ssDNA end of the DNA tether providing an 8nt toehold near the bead end of the tether (Fig. 5Aiii). In the second configuration the toehold is not subjected to force, and each step towards displacement during branch migration leads to one additional base from the incumbent strand that is not withstanding the force. We tested the strand displacement reaction rate in FLOChip by monitoring removal of the tethered beads that were subjected to forces in 0.75–10 pN range. Since bead removal events could be attributed to either strand displacement or dissociation of Dig-AntiDig binding interaction, we tested the dissociation rate of Dig-AntiDig binding interaction in the range of forces tested (0.75–10 pN) by monitoring bead removal events in the absence of the displacing strand (Supplementary Fig. S6A). The dissociation rate of Dig-AntiDig binding interaction remained ~two orders of magnitude below the rate of bead removal when the invader strand was introduced under either configuration (Strained and strain-free toehold), confirming that vast majority of bead removal events in this force range are due to displacement of the Dig-modified oligo by the invader strand (Supplementary Fig. S6B). Since Dig-AntiDig dissociation and strand displacement events occur independently, the strand displacement reaction rate can be extracted by subtracting the bead removal rate in the presence of the displacing strand from the corresponding rate in the absence of the displacing strand.

In the strained toehold configuration, we observed a modest but detectable increase in the rate of strand displacement in the 0.75–5 pN range, and the reaction rate remained unchanged in the 5–10 pN range (Fig. 5B). In the strain-free toehold configuration, the strand displacement reaction rate was independent of the mechanical load at forces <~4 pN (Fig. 5B). However, the rate of strand displacement increased by more than an order of magnitude as the force increased from 5 to 10 pN (Fig. 5B).

## Discussion

This study reports the use of FLO-Chip to simultaneously characterize up to thousands of molecular binding interactions in parallel. We focused on probing interactions that are widely used in single-molecule assays (DIG-Ant-DIG and biotin-streptavidin) and in molecular force sensing experiments (DNA-DNA unzipping). For the interactions that have been previously studied, the values for most probable rupture force and energy landscape parameters determined using FLO-Chip agreed well with previously reported values using other force spectroscopy techniques, verifying the validity of the FLO-Chip assay. Hence, FLO-Chip provides similar high-quality force characterization as other single-molecule force spectroscopy techniques while offering the significant advantages of reduced cost for each experiment (See Supplementary Table 1) along with execution of massively parallel mechanical manipulation of molecular binding interactions under multiplexed mechanical loading rates.

The multiplexing range enabled using FLO-Chip is significantly enhanced compared to the throughputs reported using magnetic tweezers^11^ optical manipulation^53^ and on-a-chip atomic force spectroscopy^54^ on multiple fronts: (i) the number of readouts per chip presented in this study are ~3 times higher than throughputs reported using centrifugal force spectroscopy^9^ and acoustic force spectroscopy^10^, (ii) we introduced the capacity to multiplex loading rates by simultaneously imaging multiple channels with varying widths enabling full characterization of molecular binding interactions in one test, (iii) the automated bead selection and rupture detection results in significantly reduced total analysis time of the force spectroscopy test images (total analysis time of ~5 min using MATLAB programming for total of ~4000 analyzed beads). Moreover, FLO-Chip leverages a simple preparation procedure prior to each force spectroscopy test (~2 hours). Hence, full mechanical characterization of a molecular interaction can be carried out in a few hours as opposed to several days or even weeks with other methods. Notably, a recent study that leverages a combination of fluid flow and magnetic tweezers report multiplexed analysis of up to ~50,000 single-molecule events^17^. A comparable level of throughput can potentially be achieved using FLOChip via the use of more-advanced imaging techniques and higher-resolution cameras that can enable imaging of the entire chip shown in Fig. 1.

We used FLOChip to investigate the effect of force on toehold-mediated strand displacement and demonstrate its utility in facilitating high-throughput studies of single-molecule interactions under a wide range of mechanical loads. The process of toehold-mediated strand displacement consists of three consecutive subprocesses: (i) Toehold binding, (ii) branch migration, and (iii) detachment of the incumbent strand, which occur in series. Therefore, the change in overall reaction rate under force is dependent on how force alters the rate of each subprocesses. We observed three main trends. First, the case where the toehold was subjected to load exhibited a modest increase in the rate of strand displacement at low forces (~0.75-5 pN) range. This outcome could potentially be due to the load stretching the ssDNA toehold to a length that facilitates docking and hybridization of the invading strand (Supplementary Fig. S7Ai)^51^. Previous studies have shown that at ~5 pN force, the amount of extension per base in ssDNA domain matches that of dsDNA^55^, suggesting the presence of a small load that stretches ssDNA might reduce the entropic cost of converting from ssDNA to dsDNA. We observed no further change in the rate of strand displacement when the strained-toehold configuration was subjected to force >5 pN. This result could be due to competing effects of >5 pN force on toehold hybridization and dissociation of the incumbent strand. Toehold hybridization would be disfavored (i.e. slowing down strand displacement) if the ssDNA toehold is stretched beyond the length of dsDNA due to the presence of force. In contrast, dissociation of the incumbent strand from the substrate would be facilitated due to the force (Supplementary Fig. S7Aiii). The force would not favor forward or reverse branch migration steps, so the process of branch migration likely is not dependent on the applied force (Supplementary Fig. S7Aii). In the strain-free toehold configuration, we observed a lack of force-dependence below ~5 pN, which is consistent with the toehold binding rate being independent of the applied force (Supplementary Fig. S7Bi). The presence of >~5 pN force led to a significant increase in the rate of strand displacement by over an order of magnitude at 10 pN, which is likely due to the force facilitating the branch migration and dissociation processes. In this configuration, each forward branch migration step results in one less base-pair that is withstanding the force, and at forces greater than ~5 pN, overextension of the newly formed ssDNA section should disfavor the rebinding of the DIG oligo, which likely facilitates progression of branch migration (Supplementary Fig. S7Bii). In addition, application of force accelerates the rate of bead detachment further enhancing the overall rate of strand displacement reaction (Supplementary Fig. S7Biii).

While recent efforts such as centrifugal force spectroscopy also reduce the cost associated with force spectroscopy^9^, FLO-Chip can further reduce the cost of mechanical characterization of molecular interactions. The use of PDMS in FLO-Chip enables significant reduction in cost of each device (~3$ per chip, see Supplementary Table 1). Furthermore, FLO-Chip relies on conventional microscopes and syringe pumps resulting in significantly reduced instrumentation cost. Further reduction in total cost can be considered if a lower-cost syringe pump is used to probe the rupture force under constant mechanical load, and the FLO-Chip readout only requires the ability to detect micron-sized beads in bright field imaging, which could likely be carried out on existing microscopes in many research laboratories. Therefore, FLO-Chip can enable single-molecule force spectroscopy tests at low cost and greater accessibility for all labs (cost details in Supplementary Table 1). It is worth noting that one needs to rely on more advanced force spectroscopy techniques such as optical tweezers if there is a need for high spatial (nanometer scale) and temporal (millisecond range) resolution^1,7^. In contrast, a distinct advantage of FLO-Chip (Fig. 4) is its massively parallel force spectroscopy readout that can be resolved using conventional laboratory cameras readily available in most labs with micrometer scale spatial and 100 ms temporal resolutions (Supplementary Fig. S8).

The compact and portable nature of the FLO-Chip system is also conducive for performing force spectroscopy in college- or possibly high school-level educational laboratories that contain conventional bright field microscopes. Furthermore, recent advancements in cellphone-based mobile microscopy^56^ could provide a route to eliminating the need for a microscope altogether, which could even further reduce barriers for research and educational laboratories. Finally, the prospect of integrating the FLO-Chip assay with cellphone-based microscopy can potentially enable in-field or point-of-care application for detection of changes in solution rheological properties, altered ion concentration, or the presence or interactions of biomarkers. Moreover, the use of microfluidics can enable integration of single-molecule force spectroscopy into the vast applications of microfluidics in biomedical and biophysical research^54,57–60^. Overall, we anticipate FLO-Chip can make single-molecule force spectroscopy a safe and easily accessible tool to a wider range of laboratories with substantially lowered cost and increased throughput.

## Methods

Extended details for materials and experimental methods are included in the Supplementary Information file.

### Flow cell preparation

The coverslip substrates were functionalized with a layer of polyethylene glycol (PEG) and biotinlated-PEG as previously described^61,62^. A 50:1 ratio between PEG and biotinlated-PEG was used to yield a uniformly coated substrate surface with biotin. The FLO-Chip microfluidic devices were fabricated using standard SU-8 photolithography and polydimethylsiloxane (PDMS) soft lithography^63^. After plasma-bonding the PDMS microdevices on the coverslips coated with PEG/Biotin-PEG, the formed channels were flushed with streptavidin and passivated with bovine serum albumin to prepare the FLO-Chip devices for each force spectroscopy test.

### Assembly of the beads tethered to the coverslip

The ~1.9 µm tether used in the force spectroscopy tests were prepared by ligating biotin-modified and single-stranded oligos to the two ends of a linearized double stranded plasmid with four base sticky ends. The tethers were purified using high-pressure liquid chromatography and preincubated with excess end oligos to anneal the desired molecular assembly to the single-stranded end of the tether. Following the annealing reaction, the tethers were added to the passivated FLO-Chip devices to allow the biotin end of the tether to bind to the streptavidin-coated coverslip surface. After washing the extra unbound tethers, anti-digoxigenin-coated microbeads were added to the FLO-Chip devices to allow beads to bind to the digoxigenin end of the tethers.

### Flow cell calibration

Controlled level of flow rate was applied using a syringe pump. To estimate the amount of force applied to the tethered beads, the lateral fluctuation of the beads under constant flow rate was monitored over time. The equipartition theorem was used to estimate the applied force based on the average mean-squared lateral fluctuations and the tether length under force as described previously^15^.

### Bead displacement analysis

The time-lapse bead images were analyzed using a custom-built MATLAB code that consists of previously reported MATLAB code^64^. Bead displacement was detected with subpixel resolution by making a Fourier transform of the bead image at each time point followed by making cross-correlation of the Fourier transform image with respect to the Fourier transform corresponding to bead image at time zero. The cross-correlated image was then submerged in an expanded matrix to achieve a desired ~10 nm resolution. We utilized a previously reported localized submerging strategy^64^ to significantly reduce the computational cost of the subpixel bead tracking analysis.

### Most probable rupture force analysis

The probability density of rupture ${{{{{\rm{p}}}}}}({{{{{\rm{F}}}}}})$ for a molecular receptor-ligand binding interaction for a given force (${{{{{\rm{F}}}}}}$) can be described as:

5 $${{{{{\rm{p}}}}}}({{{{{\rm{F}}}}}})=\frac{{{{{{{\rm{k}}}}}}}_{{{{{{\rm{off}}}}}}}}{\dot{{{{{{\rm{F}}}}}}}}{{\exp }}\left(\frac{{{{{{\rm{F}}}}}}\triangle {{{{{\rm{X}}}}}}}{{{{{{{\rm{k}}}}}}}_{{{{{{\rm{B}}}}}}}{{{{{\rm{T}}}}}}}\right){{\exp }}\left(\frac{{{{{{{\rm{k}}}}}}}_{{{{{{\rm{B}}}}}}}{{{{{\rm{T}}}}}}{{{{{{\rm{k}}}}}}}_{{{{{{\rm{off}}}}}}}}{\dot{{{{{{\rm{F}}}}}}}\triangle {{{{{\rm{X}}}}}}}\left(1-{{\exp }}\left(\frac{{{{{{\rm{F}}}}}}\triangle {{{{{\rm{X}}}}}}}{{{{{{{\rm{k}}}}}}}_{{{{{{\rm{B}}}}}}}{{{{{\rm{T}}}}}}}\right)\right)\right.$$

where ${{{{{{\rm{k}}}}}}}_{{{{{{\rm{off}}}}}}}$ is the dissociation rate at zero force, $\triangle {{{{{\rm{X}}}}}}$ is the potential width and $\dot{{{{{{\rm{F}}}}}}}$ is the applied loading rate^33,36^. The cumulative probability of rupture occurrence ${{{{{\rm{P}}}}}}({{{{{\rm{F}}}}}})$ for a given force (${{{{{\rm{F}}}}}}$) can be estimated with:

6 $${{{{{\rm{P}}}}}}({{{{{\rm{F}}}}}})={\int _{0}^{{{{{{\rm{F}}}}}}}}{{{{{\rm{p}}}}}}\left({{{{{\rm{f}}}}}}\right)\,{{{{{\rm{df}}}}}}\, \approx \left[{\sum }_{{{{{{\rm{n}}}}}}=1}^{{{{{{\rm{N}}}}}}}\frac{{{{{{{{\rm{a}}}}}}}_{1}}^{{{{{{\rm{n}}}}}}}}{{{{{{\rm{n}}}}}}!{{{{{{{\rm{a}}}}}}}_{2}}^{{{{{{\rm{n}}}}}}}}{{\exp }}\left({{{{{\rm{n}}}}}}{{{{{{\rm{a}}}}}}}_{2}{{{{{\rm{F}}}}}}\right)\right]{{\exp }}\left\{\frac{{{{{{{\rm{a}}}}}}}_{1}}{{{{{{{\rm{a}}}}}}}_{2}}(1-{{\exp }}({{{{{{\rm{a}}}}}}}_{2}{{{{{\rm{F}}}}}}))\right\}$$

where $${{{{{{\rm{a}}}}}}}_{1}=$$ $$\frac{{{{{{{\rm{k}}}}}}}_{{{{{{\rm{off}}}}}}}}{\dot{{{{{{\rm{F}}}}}}}}$$ and $${{{{{{\rm{a}}}}}}}_{2}=$$ $$\frac{\triangle {{{{{\rm{X}}}}}}}{{{{{{{\rm{k}}}}}}}_{{{{{{\rm{B}}}}}}}{{{{{\rm{T}}}}}}}$$ .

The obtained rupturing force population for each experimental condition was binned cumulatively and were fitted with Eq. 6 with the corresponding loading rate as an input to extract $\triangle {{{{{\rm{X}}}}}}$ and ${{{{{{\rm{k}}}}}}}_{{{{{{\rm{off}}}}}}}$. The estimated solution in Eq. 6 included the first 50 terms to accurately estimate the cumulative probability density function (Supplementary Fig. S4). The extracted coefficients ${{{{{{\rm{k}}}}}}}_{{{{{{\rm{off}}}}}}}$ and $\triangle {{{{{\rm{X}}}}}}$ along with the corresponding loading rate $\dot{{{{{{\rm{F}}}}}}}$ were then used to report the most probable rupture force ${{{{{{\rm{F}}}}}}}^{*}$ according to Eq. 3^36^. The most probable rupturing force for each experimental condition was reported in mean ± standard deviation format. To report the mean and standard deviation values, the rupturing force population from each experimental condition was randomly divided to 3 subgroups using random selection without replication.

### Statistics and Reproducibility

To report the mean and standard deviation values, the rupturing force population from each experimental condition was randomly divided to 3 subgroups using random selection without replication. No statistical method was used to predetermine sample size. No data were excluded from the analyses. The experiments were not randomized. The Investigators were not blinded to allocation during experiments and outcome assessment.

### Reporting summary

Further information on research design is available in the Nature Research Reporting Summary linked to this article.

## Supplementary information

Supplementary Information

Reporting Summary

## Data Availability

The raw data pertaining to all figures included in our manuscript are available on the open science framework (DOI 10.17605/OSF.IO/A6BFQ). The shared data includes time-lapse files with Nikon NIS-Elements ND2 image format. Source data are provided with this paper.

## Source data

Source Data

## Code availability

Custom Matlab code files developed for analysis of video and image data are available on github (https://github.com/cecastro39/flochip/). This custom Matlab code used to analyze our data is based on previously published code^64^.
