# Supplementary material for: Low cost and massively parallel force spectroscopy with fluid loading on a chip
Source: Nat Commun. 2022 Nov 10;13:6800. doi: 10.1038/s41467-022-34212-w (PMC9649742; doi:10.1038/s41467-022-34212-w)
Supplement: Supplementary file 1 — Supplementary Information [file 41467_2022_34212_MOESM1_ESM.pdf]

Supplementary Information

Low Cost and Massively Parallel Force Spectroscopy with Fluid Loading on a Chip

Ehsan Akbari[1], Melika Shahhosseini[1], Ariel Robbins[2,3], Michael G. Poirier[2,3], Jonathan W. Song[1,4], Carlos E. Castro*[1,2]

[1]Department of Mechanical and Aerospace Engineering, The Ohio State University, Columbus, OH, 43210, USA

[2]Biophysics Graduate Program, The Ohio State University, Columbus, OH, 43210, USA

[3]Department of Physics, The Ohio State University, Columbus, OH, 43210, USA

[4]Comprehensive Cancer Center, The Ohio State University, Columbus, OH, 43210, USA

* correspondence:

Prof. Carlos E. Castro

Department of Mechanical and Aerospace Engineering

The Ohio State University

E328 Scott Laboratory, Peter L & C

201 W. 19th Ave, Columbus, OH 43210

email: castro.39@osu.edu

1. Supplementary Methods

Coverslip functionalization with Biotin-polyethylene glycol (PEG)

The coverslip functionalization with biotin-PEG was performed by piranha cleaning coverslips followed by aminosilanization and reaction with PEG which included a small fraction of biotin-PEG as previously reported[1, 2]. Briefly, coverslips were immersed in piranha solution for 30 min (20% H_2O_2 (Sigma Aldrich) and 80% concentrated H_2SO_4 (Sigma Aldrich)) followed by thorough rinsing with water (Milli-Q) (3X). To ensure that the coverslip surface is water-free, the coverslips were then dried overnight at 65C prior to the silane reaction. Prior to aminosilanization, the coverslips were swirled in Acetone (Sigma) for 10min followed by slow addition of (3-aminopropyl) triethoxysilane to final concentration of 2% vol/vol. After 2min of silane reaction, the coverslips were immersed in 1:1 vol/vol mixture of acetone and water to quench the silane reaction followed by thorough rinsing with water (Milli-Q) (3X). The coverslips were then dried overnight at 65C prior to pegylation.

To pegylate the coverslips, a solution containing 10% wt/vol mPEG (5kDa, Sigma Aldrich) and 0.2% wt/vol Biotin-mPEG (Sigma Aldrich) was prepared in $K_2B_4O_7$ (Sigma Aldrich) was prepared to enable 50:1 w/w ratio between mPEG and Biotin-mPEG. The silanized coverslips were incubated with the prepared mPEG/Biotin-mPEG solution for 90 min followed by thorough rinsing with water (Milli-Q) (3X) to remove the excess PEG. Next, the coverslips were dried with dry N_2 and stored in room temperature.

Microfluidic Flow Cell Preparation

The microfluidic platform was fabricated using SU-8 photolithography and polydimethylsiloxane (PDMS) soft lithography[3]. Briefly, SU-8 2050 was spin-coated on a 4in Silicon wafer (University Wafers) followed by UV exposure through a transparency mask and development to replicate the designed microfluidic patterns. The Silicon wafer

with the fabricated monolithic features (120μm in height) was then used to cast the microchannels in PDMS. Following PDMS development, individual flow cells were cut and plasma-bonded on the coverslips functionalized with mPEG/Biotin-mPEG to form the flow cell.

Following the formation of the flow cells, the channels were flushed with 0.1mg/mL Streptavidin (Sigma Aldrich) in PBS (Fisher Scientific) and incubated for 10min to enable binding between free streptavidin and the Biotin-mPEG on the coverslip surface. The flow cells were flushed with PBS to remove the streptavidin excess. Next, the flow cells were flushed with blocking buffer (1mg/mL BSA (Life Technologies), 5mM Tris (Sigma Aldrich), 5mM NaCl (Sigma Aldrich), 1mM EDTA (Sigma Aldrich), 3mM NaN_3 (Sigma Aldrich), 0.1% vol/vol Tween-20 (Sigma Aldrich)) and incubated for 30 to reduce nonspecific interactions with the coverslip surface. Next, the coverslips were flushed with experimental buffer (5mM Tris (Sigma Aldrich), 5mM NaCl (Sigma Aldrich), 1mM EDTA (Sigma Aldrich), 0.1% vol/vol Tween-20, 5mM $MgCl_2$ (Sigma Aldrich)) to remove the excess blocking buffer prior to each force spectroscopy experiment.

Tether Construct Preparation

Tether preparation starts with the digestion of a ~2 μm double stranded plasmid by the BsaI restriction enzyme (NEB R0535S) in 1x CutSmart Buffer (NEB B7204S). Depending on the age of the enzyme, 1-3 Units of BsaI per μg of plasmid in a 10 uL reaction volume is usually sufficient to cut the plasmid without over-digestion. The incubation is carried out at 37C for 60 minutes followed by enzyme inactivation at 65C for 20 minutes. This enzyme cuts the plasmid downstream of its recognition site, leaving a four base single stranded overhang. For each end of the tether, a pair of oligos pre-

annealed at room temperature for 15 minutes in 50 mM NaCl can be ligated on using T4 DNA Ligase (NEB M0202S) in 1x T4 ligase buffer (NEB B0202S). In order to facilitate ligation, the oligo containing the 5' end that will ligate to the BsaI cut plasmid end must be kinased using T4 Polynucleotide kinase (PNK) (NEB M0201S). Incubation is carried out with a T4 PNK concentration of 10 Units per 300 pmole ends for 90 minutes at 37C, then 20 minutes at 65C. Because the oligos will be subsequently ligated, we carry out the reaction in 1x T4 ligase buffer instead of T4 PNK buffer. One oligo pair facilitates attachment to the slide via a biotin-streptavidin-biotin connection and the other pair contains a 30 nt overhang. The ligation is carried out with a final enzyme reaction concentration of 4 Units/μL, DNA concentration of 200nM, and 100-fold excess of both pairs of oligo ends (compared to the linearized plasmid). It is incubated for 30 minutes at room temperature followed by a heat shock at 65C for 25 min. After the ligation, EDTA is added to make a final concentration of 20mM in order to chelate excess magnesium in case any ligase survived the heat shock.

Before HPLC purification, the sample must go through a phenol-chloroform extraction to remove any proteins, such as BSA, that have the potential to stick to the HPLC column. HPLC purification is carried out on a Gen-Pak column (Waters WAT015490) with a salt ramp going from TE100 (25mM Tris-HCL, 1mM EDTA, 100mM NaCl) to TE1000 (25mM Tris-HCL, 1mM EDTA, 1M NaCl) to remove the excess oligo ends from the final tether product. A small amount of the fractions collected from the HPLC are then run on a 0.7% agarose gel at 225 volts for 35 minutes, then post-stained with Ethidium Bromide. This ensures we got good separation of the excess oligo ends from the final tether product and confirms what fractions to keep. The remaining amount

of the fractions containing the desired final product are then concentrated and buffer exchanged into 0.5X TE for a total of 3 times using a 30K amicon centrifuge filter (Sigma UFC203024). The final concentration of the tether is then determined via the 260nm absorbance peak on a spectrophotometer.

Assembly of Beads tethered to the coverslip via a single DNA tether

DNA tethers were preincubated at 200pM with excess end oligos at 45C for 1hour followed by incubation at 37C for 1 hour prior to each experiment. For the experiments involving dissociation of Biotin-Streptavidin and Digoxigenin-Anti Digoxigenin, the Biotin-ssDNA end oligo was added at 100X excess to the tether. For the experiment involving unzipping of double stranded DNA, the Cy3-ssDNA was added at 100X excess, and the Dig-ssDNA and Cy5-ssDNA were added at 200X.

Following the incubation of tether with end oligos, the flow cells were flushed with the tether solution and incubated for 30min at room temperature to enable anchoring of the tether to the coverslip through binding between streptavidin on surface and biotin on the tether end. Next, the flow cells were flushed with the experimental buffer to remove the excess tether followed by addition of the beads. The beads were added at 0.1wt/vol concentration with 10% blocking buffer and 5mM $MgCl_2$. The 10% blocking buffer was added to reduce bead aggregation and non-specific interaction between the surface and the beads during the experiments.

Flow Application

The flow cell was connected to a 2.5mL Harvard Syringe 1000 (Fisher Scientific) via translucent tubes with 0.8mm inner diameter (Cole-Parmer). The syringe was

connected to a programmable syringe pump (Harvard Apparatus) to apply flow within the microchannels. Prior to start of the experiment, the beads were allowed to bind to the free end of the anchored tethers for 15min under static condition. Next, the excess beads were washed using a small flow rate prior to the start of each experiment. To apply controlled levels of loading rate on the studied molecular interactions, the beads were subjected to a linear flow ramp programmed using the syringe pump. The slope of the ramp was controlled in order to produce a desired loading rate.

Flow Cell Calibration

We calibrated the flow cells using equipartition theorem as previously described[4]. We performed calibration in the microchannel with largest width (2500μm in width × 120μm in height), and the obtained calibration chart was interpolated to the microchannels according to the channel width. In order to determine the location of the base of the DNA tether through which each bead is anchored to the coverslip, we monitored the bead center while subjecting the beads to 100 μLmin^{-1} backward flow followed by 100 μLmin^{-1} forward flow. These flow rates were chosen to enable full stretching of the 5745nt dsDNA tethers. Subjection to these flow rates results in equivalent stretching of the DNA tether in each direction. Therefore, the midpoint along the line that connects the bead center position recorded under stretched condition in each direction denotes the location at which the tethers are anchored to the coverslip (Fig. S1). Next, the beads were subjected to a series of flow rates ranging from 0.25 μLmin^{-1} to 1.0 μLmin^{-1}. The beads were kept subject to each flow rate for 2min to record the average mean-squared lateral fluctuation of the center of the bead ($< \delta y^2 >$) along with the length of the stretched tether (l). The beads were imaged at 50Hz to extract an accurate mean

square lateral displacement of the bead center. Moreover, the end-to-end length of the stretched tether (l) was obtained based on averaged displacement of bead center along the flow direction and the bead radius (r) (Fig. 1Di). According to the equipartition theorem, the stretching force applied on the tether (F) can be defined as:

$$F = \frac{k_B T \ (l+r)}{<\delta y^2>} \ (1)$$

where k_B is the Boltzmann constant and T is temperature[5, 6]. This equation suggest that the level transverse fluctuations of the bead center decrease when the bead is subjected to higher forces (Fig S2). We performed the calibration and all the test experiments at room temperature (23 C) correlating to $k_B T$=4.114 pN.nm.

Image Acquisition and Analysis

Bright field imaging was performed using a Nikon TiE with Nikon NIS Elements software (Nikon NIS Elements Advanced Research). Perfect focus was used to maintain the beads in focus during the course of each measurement. For the experiments pertaining calibration, the beads were imaged using a 100X oil immersion objective (80μm×80μm imaging filed). All images were analyzed using a Custom-Built MATLAB (R2021a) code. The epifluorescence images of the beads obtained from the time-lapse epifluorescence imaging files were first converted to binary format to detect the location of each bead. Bead displacement was detected with subpixel resolution by making a Fourier transform of the bead image at each time point followed by making cross-correlation of the Fourier transform image with respect to the Fourier transform corresponding to bead image at time zero. The cross-correlated image was then submerged in an expanded matrix to achieve a desired ~10nm resolution. The

implemented autocorrelation subpixel analysis has enabled accurate monitoring of ~10nm range and ~1nm range bead displacement in magnetic tweezers[11] and optical traps[12], respectively.

An inverse Fourier Transform was then performed on the submerged matrix. The row and column index corresponding to the overall maxima of the inverse Fourier transform matrix denotes the subpixel in-plane displacement of the bead with respect to time zero. Since performing the inverse Fourier Transform on the larger submerged matrix requires a high computational cost, we utilized a previously reported localized submerging strategy[7] to significantly reduce the computational cost of the subpixel bead tracking analysis.

For the force spectroscopy experiments, the beads were imaged using a 40X oil immersion objective (200μm×200μm imaging filed). The time of each rupturing event was recorded manually when a bead anchored through a tether was detached and flushed away. The recorded time was then used along with the applied loading rate to extract the force at which each molecular interaction ruptures. The beads that did not displace significantly (~2μm) were detected as non-specific interactions and were excluded from the analysis. For massively parallel force spectroscopy tests under multiplexed mechanical loading rates, the chip was imaged using a 4X air objective on Nikon TS-100 (1660μm×2200μm imaging filed). Beads were subjected to two opposite flow rates to stretch the DNA tether in each direction. Beads that displaced significantly (greater than 2 μm) were automatically selected using a custom-built MATLAB code. Rupture flow rate for each bead was estimated by converting the bright-field time lapse images to binary format and monitoring the detected bead area. The flow rate at which each bead is first

undetected is marked as the corresponding rupture flow rate using a custom-built MATLAB code. This MATLAB code is based on a combination of previously published publicly available code [7] .

Data Analysis

The probability density of rupture $p(F)$ for a molecular receptor-ligand binding interaction for a given force (F) can be described as:

$$p(F) = \frac{k_{off}}{\dot{F}} exp\left(\frac{F\Delta X}{k_B T}\right) exp\left(\frac{k_B T k_{off}}{\dot{F}\Delta X}(1 - exp\left(\frac{F\Delta X}{k_B T}\right)\right) \quad (2)$$

where k_{off} is the dissociation rate at zero force, ΔX is the potential width and $\dot{F}$ is the applied loading rate[8, 9]. The cumulative probability of rupture occurrence $P(F)$ for a given force (F) can be estimated with:

$$P(F) = \int_0^F p(f)\ df \approx \left[\sum_{n=1}^{N} \frac{{a_1}^n}{n!{a_2}^n} exp(na_2 F)\right] exp\left\{\frac{a_1}{a_2}(1 - exp\ (a_2 F))\right\} \quad (3)$$

where $a_1 = \frac{k_{off}}{\dot{F}}$ and $a_2 = \frac{\Delta X}{k_B T}$.

The obtained rupturing force population for each experimental condition was binned cumulatively to plot cumulative rupture probability histograms with respect to applied force. The cumulative probability histograms were fitted with equation 3 with the corresponding loading rate as an input to extract ΔX and k_{off}. For each fit, the estimated solution in equation 3 including the first 50 terms were used to accurately estimate the cumulative probability density function (Fig S4). The extracted coefficients k_{off} and ΔX along with the corresponding loading rate $\dot{F}$ were then used to report the most probable rupture force F^* according to: [9]

$$F^* = \frac{k_BT}{\Delta X} \ln \frac{\dot{F}\Delta X}{k_BT.k_{off}} \text{ (4)}$$

The extracted F^* were plotted in a force versus logarithmic loading rate diagram. Performing linear fit on the F^* versus logarithmic loading rate diagrams was used to extract ΔX and k_{off} according to equation 4.

For N bonds connected serially, the apparent most probable rupture force F^* for uncooperative rupture can be described as:

$$F^* = \frac{k_BT}{\Delta X} \ln \frac{\dot{F}\Delta X}{N.k_BT.k_{off}} \text{ (5)}$$

Thus, the apparent rupture force is slightly weakened by $\frac{k_BT}{\Delta X} \ln N$ compared to the strength of a single binding interaction.

Statistical Analysis

The most probable rupturing force for each experimental condition was reported in mean ± standard deviation format. To report statistical analysis, the rupturing force population from each experimental condition was randomly divided to 3 subgroups using random selection without replication. Each subgroup population was then binned cumulatively and fitted with Eq. 3 using lead-squared curve fitting to report three most probable rupturing force values for each experimental condition. The three obtained force values were then used to report statistically averaged most probable rupturing force values for each experimental condition.

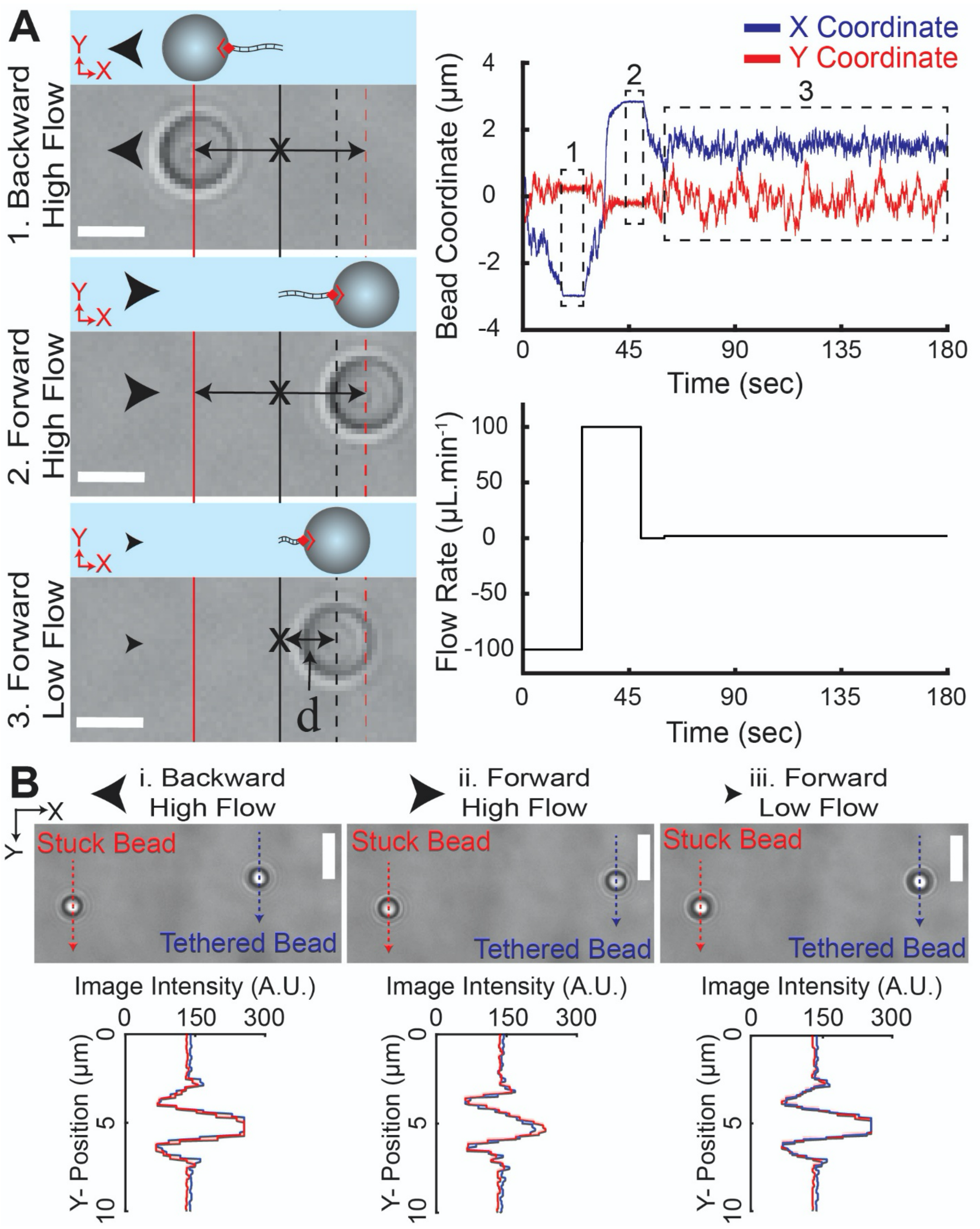
A
1. Backward High Flow
2. Forward High Flow
3. Forward Low Flow
Y
X
d
X Coordinate
Y Coordinate
Bead Coordinate (μm)
Time (sec)
Flow Rate (μL.min-1)
1
2
3
B
i. Backward High Flow
ii. Forward High Flow
iii. Forward Low Flow
Stuck Bead
Tethered Bead
Image Intensity (A.U.)
Y- Position (μm)

Supplementary Figure S1 – A) Monitoring of bead displacement under different flow conditions (black arrow heads) and extraction of tether to coverslip anchoring location (black cross). To extract the location at which the tether is anchored to the coverslip, the tethered beads were subjected to 100μL/min flow rate in each direction. The tether to coverslip anchoring location can be extracted by locating the midpoint of the line connecting the bead center (red solid and dash lines) when subjected to each flow condition (flow conditions 1 and 2). Next, the beads were subjected to slow flow (0.5μL/min shown here) for 2min (flow condition 3). Distance bead the average bead center (black dash line) and the tether to coverslip anchoring location (black solid line) denoted as d was used to extract the tether end-to-end distance (*l*). Moreover, the transverse displacement of the bead center (Y coordinate bead displacement) was used along with tether end-to-end distance to estimate the stretching force according to the equipartition theorem. Scale bars, 2μm. B) Representative images of a tethered bead next to a stuck bead subjected to different flow rates. Furthermore, the pixel intensity along the circumferential ring patterns around each bead was plotted for the tethered bead next to the stuck bead. Vertical bead movement along the z-direction results in detectable changes in circumferential ring patterns in the bead image.[10] No significant off-plane displacement of the tethered bead was detected compared to the stuck bead. Scale bars, 5μm. Source data are provided as a Source Data file.

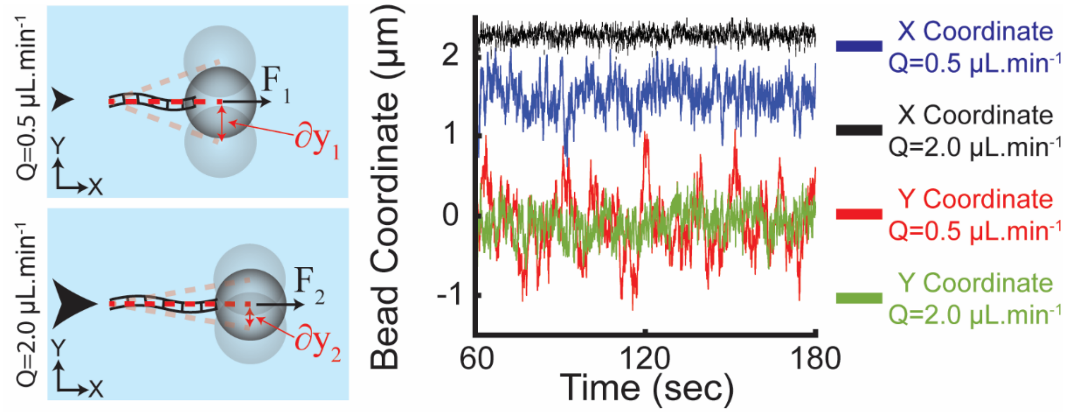

Supplementary Figure S2 – Monitoring the bead transverse displacement when subjected to different levels of flow rates. Increase in flow rate leading to increase in stretching force (F) caused increase in bead displacement in the direction of the flow (X direction) as expected. Moreover, increase in the stretching force resulted in less transverse fluctuation of the bead center (Y direction) as anticipated based on the equipartition theorem. Black arrow heads denote flow direction. Source data are provided as a Source Data file.

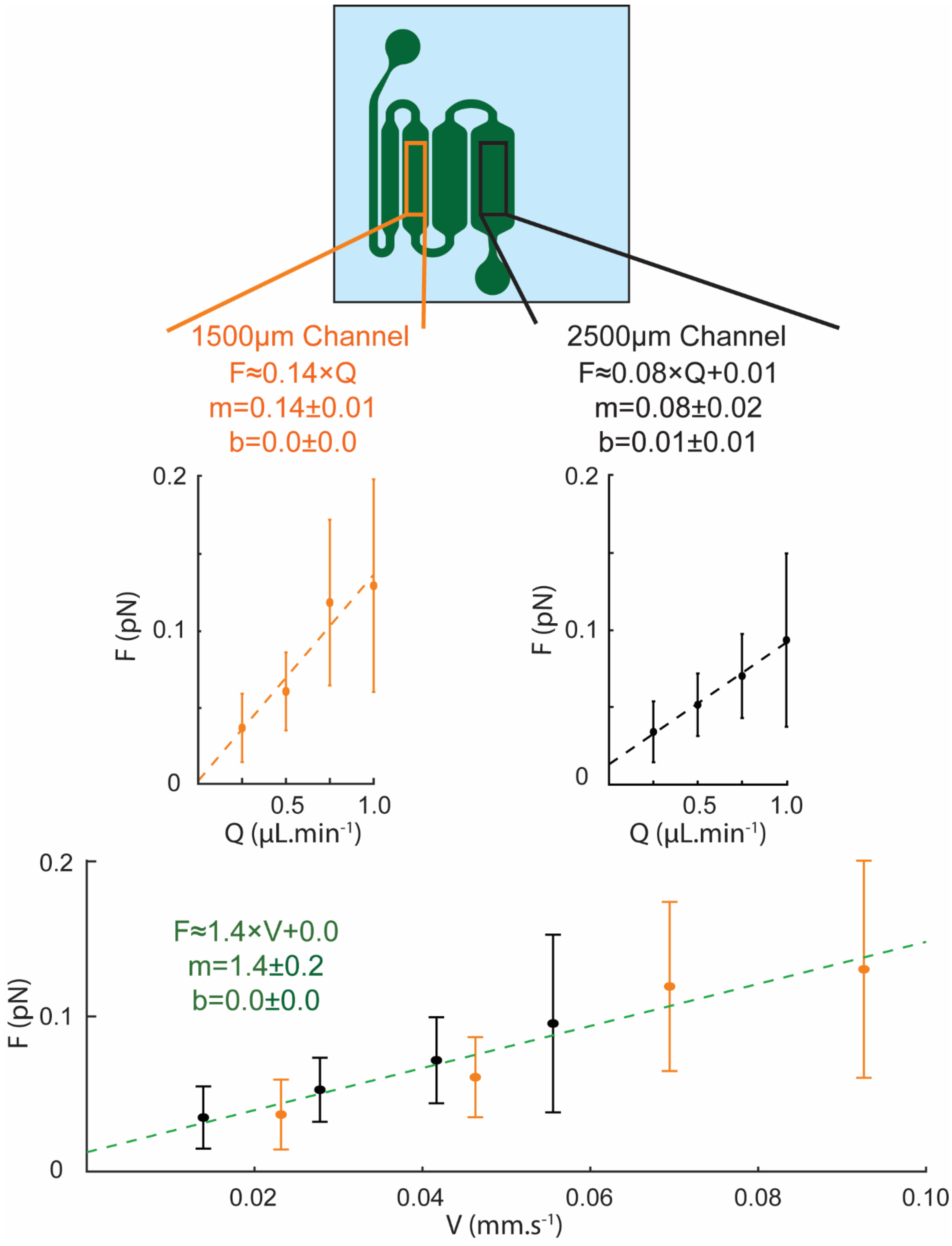
1500µm Channel
F≈0.14×Q
m=0.14±0.01
b=0.0±0.0
2500µm Channel
F≈0.08×Q+0.01
m=0.08±0.02
b=0.01±0.01
F (pN)
Q (µL.min-1)
0.2
0.1
0
0.5
1.0
F≈1.4×V+0.0
m=1.4±0.2
b=0.0±0.0
0.02
0.04
0.06
0.08
0.10
V (mm.s-1)

Supplementary Figure S3 – Calibration of stretching force when beads are subjected to different flow rates performed in two of the microchannels (Width of 1500 µm and 2500 µm). Transverse fluctuation of n=33 beads in the 2500µm wide channel and n=23 beads in the 1500µm wide channel were monitored over 3 independent experiments under different flow rates. The estimated force values are presented as mean values +/- SD. Source data are provided as a Source Data file.

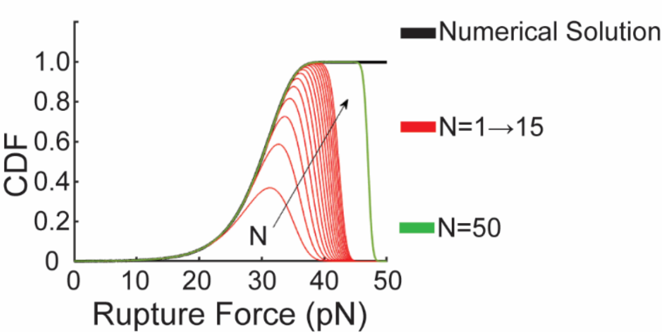

$$P(F) \approx \left[\sum_{n=1}^{N} \frac{{a_1}^n}{n!{a_2}^n} exp(na_2F)\right] exp\left\{\frac{a_1}{a_2}(1 - exp\,(a_2F))\right\}$$

Supplementary Figure S4 – Approximation of the cumulative probability density function. Including more terms in the series brings the approximate solution closer to the numerical solution. The first 50 terms (green line) was used to fit the cumulative probability density function to the force spectroscopy data.

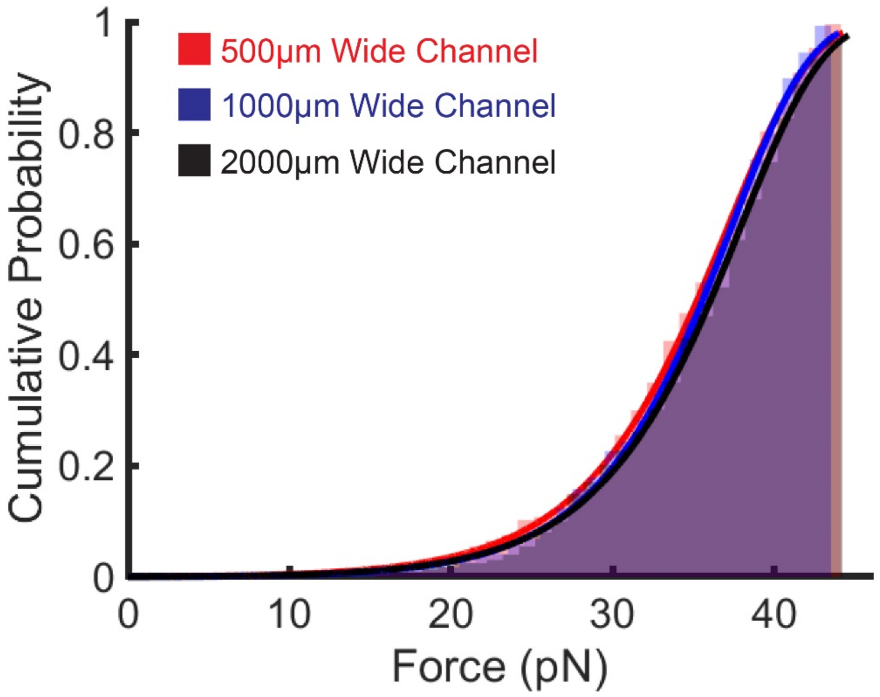

Supplementary Figure S5 - Cumulative probability histogram of the recorded DIG-AntiDIG rupture forces under 5 pN.s^{-1} in three different FLOChip channels. Source data are provided as a Source Data file.

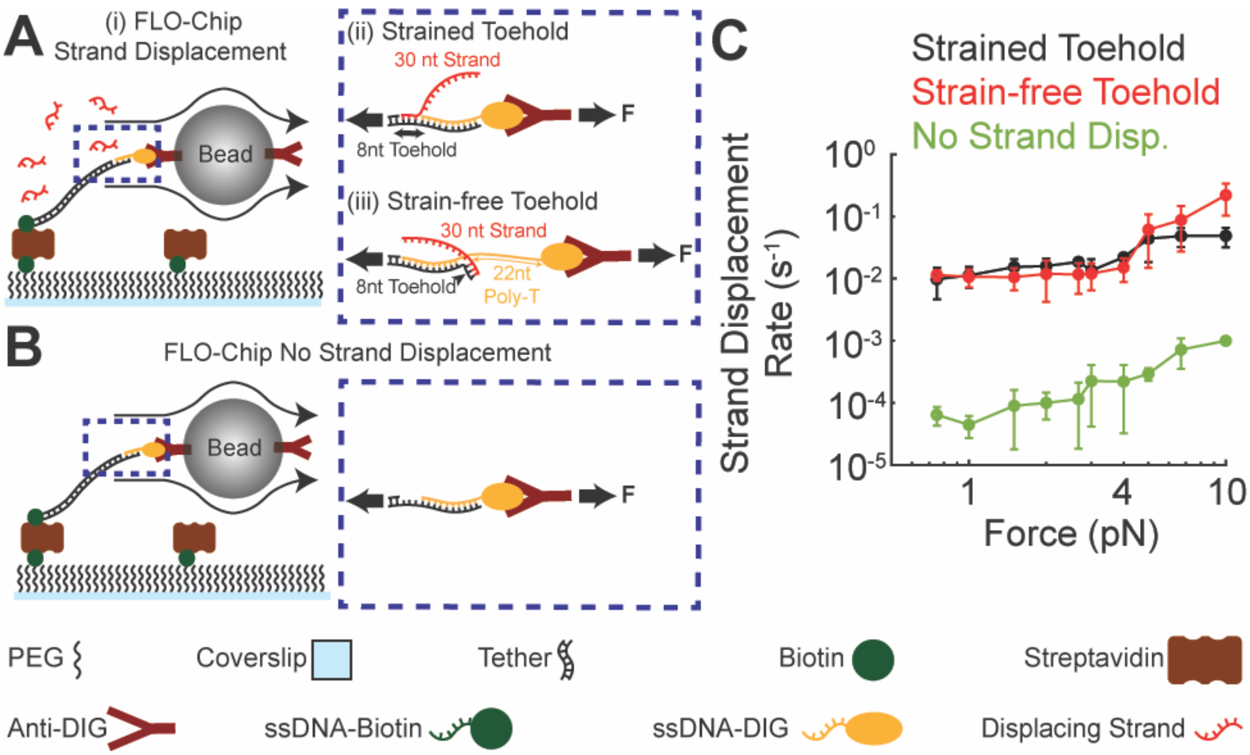

Supplementary Figure S6 – Comparison between bead removal due to strand displacement compared to DIG-AntiDIG dissociation. (A) Schematic of strand displacement in FLOChip under (ii) Strained toehold and (iii) Strain-free toehold configurations. (B) Schematic of the control configuration to monitor bead removal due to Dig-AntiDig dissociation in the absence of strand displacement. (C) Comparison between strand displacement reaction rates (Strained toehold and strain-free toehold) and Dig-AntiDig dissociation rate under force. N=3 strand displacement rates were estimated based on random selection without replication of the recorded dissociation kinetics data under each mechanical load. The estimated strand displacement rate values are presented as mean values +/- SD. Dig-AntiDig dissociation rate remains ~2 orders of magnitude slower than rate of strand displacement. Source data are provided as a Source Data file.

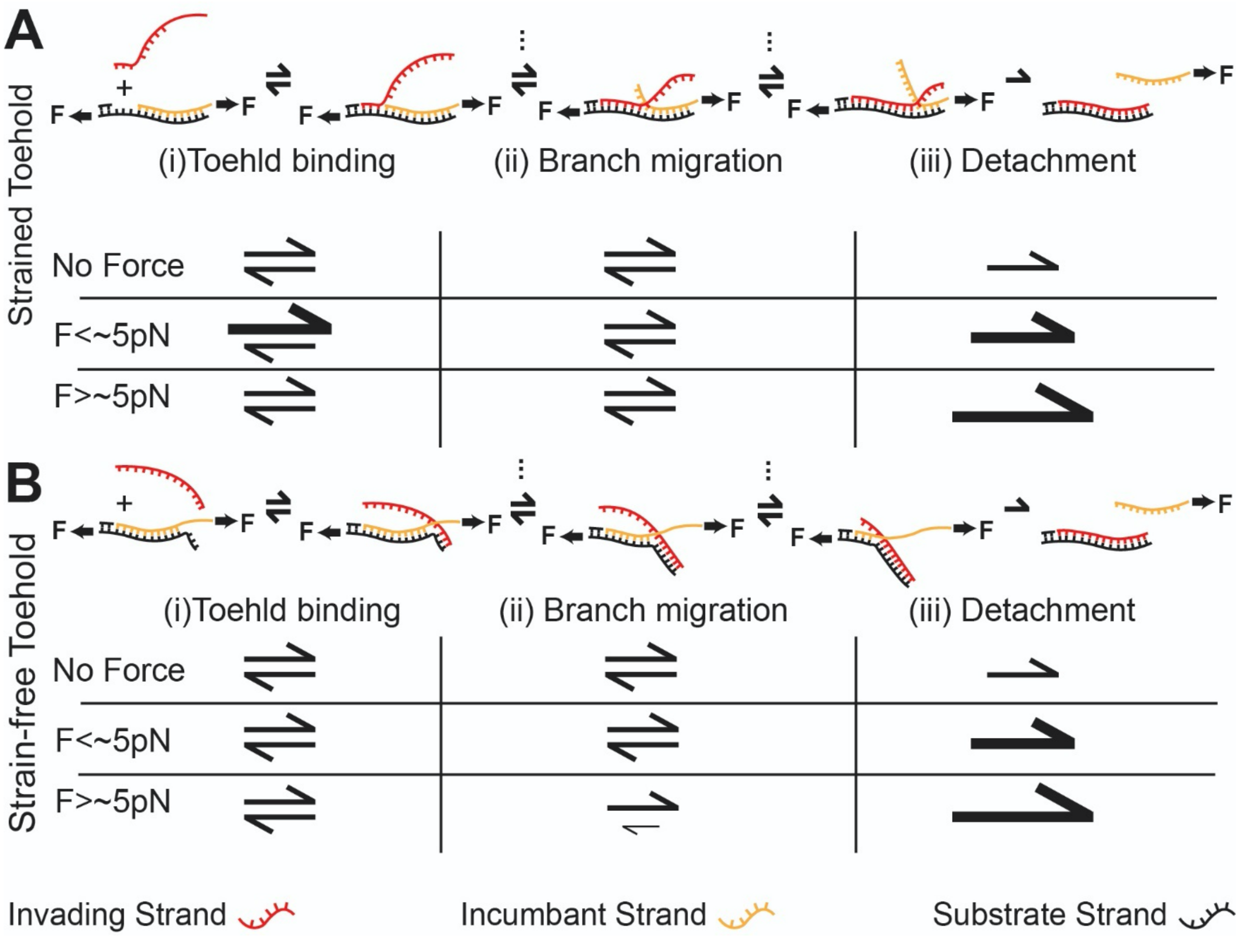

Supplementary Figure S7- Schematic illustration of the three main phases of toehold-mediated strand displacement consisting of (i) toehold binding, (ii) branch migration and (iii) detachment of the incumbent strand. (A) Schematic representation of effect of force on toehold-mediated strand displacement rate when the toehold is subjected to force. While forces smaller than ~5pN facilitate toehold binding, forces greater than ~5pN disfavor toehold binding. (B) Schematic representation of effect of force on toehold-mediated strand displacement rate when the toehold remains force-free. Forces greater than ~5pN facilitate forward branch migration compared to rebinding of

the incumbent strand. For both configurations, application of force facilitates detachment of the incumbent strand.

	Optical Tweezers	Atomic Force Microscopy	Magnetic Tweezers	Centrifugal Force Microscopy	Acoustic Force Spectroscopy	Hydrodynamic Force Spectroscopy (Single straight channel)	FLOChip
Spatial Resolution(nm)	0.1-2	0.5-1	5-10	$10\text{-}10^3$	$10\text{-}10^3$	$10\text{-}10^3$	$10\text{-}10^3$
Temporal Resolution(ms)	10^{-1}	1	$10\text{-}10^2$	$10\text{-}10^2$	$10\text{-}10^2$	$10\text{-}10^2$	$10\text{-}10^2$
Force Range (pN)*	$\sim10^{-1}\text{-}10^2$	$\sim10\text{-}10^4$	$\sim10^{-3}\text{-}10^2$	$\sim10^{-1}\text{-}10^2$	$\sim10^{-1}\text{-}10^2$	$\sim10^{-1}\text{-}10^2$	$\sim10^{-1}\text{-}10^2$
Measurement Multiplexing	No	No	Yes**	Yes	Yes	Yes	Yes
Throughput per measurement	1	1	$1\text{-}10^3$	$\sim10^3$	$\sim10^3$	$10^3\text{-}10^4$	$10^3\text{-}10^4$
Force Multiplexing	No	No	No	No	No	No	Yes
Requires Force/Stiffness Calibration	Yes	Yes	Yes	No	Yes	Yes	Yes

Supplementary Figure S8 – Comparing advantages and limitations of FLOChip with respect to other single molecule force spectroscopy techniques. *The information pertaining to optical tweezers, atomic force microscopy and magnetic tweezers is reported based on review paper by Keir C Neuman et al. [11]. *The reported values reflect the typical force ranges often achieved using each force spectroscopy technique. Forces higher than 100pN using optical tweezers[12] and up to 1nN using magnetic tweezers[13] have been demonstrated. **Parallel magnetic tweezers have been demonstrated by Iwijn De Vlaminck et al. [14].*

Item	Cost Type	Vendor	N*	Cost $
Silicon Wafer	Fixed	University Wafer Inc	NA	9.5
Cleanroom Access	Fixed	Nanotech West Lab	NA	60.0
Spin Coater	Fixed	Nanotech West Lab	NA	6.5
Contact Aligner	Fixed	Nanotech West Lab	NA	23.1
Transparency Mask	Fixed	CAD/Art Services	NA	94.0
SU-8 2100, 500mL	Fixed	Kayaku Advanced Materials	~25	760.0
**Total Fixed Cost of Photolithography**				**223.5**
Silicon Elastomer Kit, 3.9kG	Variable	Ellsworth Adhesives	~1170**	435.5
**Total Variable Fabrication Cost per Chip**				**~0.4**
Syringe Pump	Fixed	Harvard Apparatus	NA	3093.0
Hamilton Glass Syringe	Fixed	Hamilton	NA	50.4
Hamilton Needle	Fixed	Hamilton	NA	8.7

Perfusion Tubing, 25ft	Fixed	Cole-Parmer	~10	27.8
Elbow-Connector	Fixed	Cole-Parmer	NA	1.8
T-Connector	Fixed	Cole-Parmer	NA	1.5
1.5mm Biopsy Punch	Fixed	Integra	NA	3.1
4mm Biopsy Punch	Fixed	Integra	NA	3.6
**Total Fixed Cost of the Perfusion Setup**				**3164.9**
Glass Coverslip	Variable	Fisher Scientific	~66	37.3
Sulfuric Acid, 95-98%, 1L	Variable	Sigma	~200	79.7
Hydrogen Peroxide, 50% wt, 500mL	Variable	Sigma	~400	79.7
Acetone, HPLC Grade, 1L	Variable		~120	101.0
AMINOPROPYLTRIETHOXYSILANE, 99%, 100mL	Variable	Sigma	~250	78.2
5000 mPEG	Variable	Laysan Bio	~600	182.0
5000 Biotin-mPEG, 1G	Variable	Laysan Bio	~6000	195.0
**Total Variable Cost of Coverslip functionalization**				**~2.7**
**FLO-Chip Total Fixed Cost**				**~3388.4**
**FLO-Chip Total Variable Cost per Chip**				**~3.1**

Supplementary Table 1 – Cost analysis of FLO-Chip. *: N denotes the number of corresponding units that can be manufactured or synthesized with the corresponding item or regent. The numbers are estimated based on the protocols developed in the lab. **: Each silicon master fabricated in the cleanroom includes 9 chips per wafer. Furthermore,

~30mg of Silicon elastomer solution is needed for each round of soft lithography. Each purchased kit contains 3.9Kg of Silicon elastomer which enables ~130 rounds of PDMS soft lithography, enabling fabrication of ~1170 PDMS chips. The syringe pump cost accounts for the majority of the total fixed cost. The relatively high cost of the syringe pump used in this study is due to its programmability. However, much simpler types of syringe pumps with cost ranges down to ~$300 can be used depending on the application lowering the total fixed cost to ~$600. Furthermore, simpler methods of flow application such as a hydrostatic fluid column can be used for applications such as force-clamp force spectroscopy to totally eliminate the cost of the pump.

**Oligo**	**Sequence**
Tether single stranded end	CGGTCGAGCTATTGAAAGCTAGCTAGTTGTCCTTGT CTACCTGGGTGCGCACGA
Tether single stranded end complementary	AGCTAGCTTTCAATAGCTCG
Tether Biotin end	/5BiotinTEG/CCTACACTGGGATAATTGAC
Tether Biotin end Complementary	ACCGGTCAATTATCCCAGTGTAGG
ssDNA-Biotin	/5BiotinTEG/TTTTTTTCGTGCGCACCCAGGTAGACAA GGACAACT
ssDNA-DIG	/5DIG/TTTTTTTCGTGCGCACCCAGGTAGACAAGGAC AACT
18nt DNA Unzipping 33% GC Green Oligo	TATCAACAGTGAACCATATTTTCGTGCGCACCCAGGT AGACAAGGACAACT

18nt DNA Unzipping 58% GC Green Oligo	AGCCAGCAGAGACACACGTTTTCGTGCGCACCCAGGTAGACAAGGACAACT
18nt DNA Unzipping 83% GC Green Oligo	GGCCCGCAGCGACCACCCTTTTCGTGCGCACCCAGGTAGACAAGGACAACT
18nt DNA Unzipping 33% GC Gold Oligo	/5DIG/TTTTTTCTCTGGTTAACGTGTCTGGGCATTTTATGGTTCACTGTTGATA
18nt DNA Unzipping 58% GC Gold DIG Oligo	/5DIG/TTTTTTCTCTGGTTAACGTGTCTGGGCATTTCGTGTGTCTCTGCTGGCT
18nt DNA Unzipping 83% GC Gold DIG Oligo	5'dig/TTTTTTCTCTGGTTAACGTGTCTGGGCATTTGGGTGGTCGCTGCGGGCC
9nt DNA Unzipping Green Oligo	CGCGCGTACTTTTCGTGCGCACCCAGGTAGACAAGGACAACT
9nt DNA Unzipping Gold DIG Oligo	/5DIG/TTTTTTCTCTGGTTAACGTGTCTGGGCATTTGTACGCGCG
ssDNA-DIG Complementary Red Oligo	TGCCCAGACACGTTAACCAGAG

Supplementary Table 2 - List of oligos detailing the sequences.

References

1. Ha T*, et al.* Initiation and re-initiation of DNA unwinding by the Escherichia coli Rep helicase. *Nature* **419**, 638-641 (2002).

2. Roy R, Hohng S, Ha T. A practical guide to single-molecule FRET. *Nat Methods* **5**, 507-516 (2008).

3. McDonald JC*, et al.* Fabrication of microfluidic systems in poly (dimethylsiloxane). *ELECTROPHORESIS: An International Journal* **21**, 27-40 (2000).

4. Kim S, Blainey PC, Schroeder CM, Xie XS. Multiplexed single-molecule assay for enzymatic activity on flow-stretched DNA. *Nat Methods* **4**, 397-399 (2007).

5. Strick TR, Allemand JF, Bensimon D, Bensimon A, Croquette V. The elasticity of a single supercoiled DNA molecule. *Science* **271**, 1835-1837 (1996).

6. Strick T, Allemand J-F, Bensimon D, Croquette V. Behavior of supercoiled DNA. *Biophys J* **74**, 2016-2028 (1998).

7. Guizar-Sicairos M, Thurman ST, Fienup JR. Efficient subpixel image registration algorithms. *Optics letters* **33**, 156-158 (2008).

8. Evans E, Ritchie K. Dynamic strength of molecular adhesion bonds. *Biophys J* **72**, 1541-1555 (1997).

9. Evans E. Probing the relation between force—lifetime—and chemistry in single molecular bonds. *Annual review of biophysics and biomolecular structure* **30**, 105-128 (2001).

10. Gosse C, Croquette V. Magnetic tweezers: micromanipulation and force measurement at the molecular level. *Biophysical journal* **82**, 3314-3329 (2002).

11. Neuman KC, Nagy A. Single-molecule force spectroscopy: optical tweezers, magnetic tweezers and atomic force microscopy. *Nature methods* **5**, 491-505 (2008).

12. Dong J, Castro CE, Boyce MC, Lang MJ, Lindquist S. Optical trapping with high forces reveals unexpected behaviors of prion fibrils. *Nature structural & molecular biology* **17**, 1422-1430 (2010).

13. Kollmannsberger P, Fabry B. BaHigh-force magnetic tweezers with force feedback for biological applications. *Review of Scientific Instruments* **78**, 114301 (2007).

14. De Vlaminck I*, et al.* Highly parallel magnetic tweezers by targeted DNA tethering. *Nano letters* **11**, 5489-5493 (2011).
