# Supplementary material for: Low cost and massively parallel force spectroscopy with fluid loading on a chip
Source: Nat Commun. 2022 Nov 10;13:6800. doi: 10.1038/s41467-022-34212-w (PMC9649742; doi:10.1038/s41467-022-34212-w)
Supplement: Supplementary file 2 — Reporting Summary [file 41467_2022_34212_MOESM2_ESM.pdf]

nature portfolio

Corresponding author(s): Double-blind peer review submissions: write DBP[illegible] Carlos Castro [illegible]re inste[illegible]

Last updated by author(s): YY[illegible] 08/01/2022

Reporting Summary

Nature Portfolio wishes to improve the reproducibility of the work that we publish. This form provides structure for consistency and transparency in reporting. For further information on Nature Portfolio policies, see our Editorial Policies and the Editorial Policy Checklist.

Statistics

For all statistical analyses, confirm that the following items are present in the figure legend, table legend, main text, or Methods section.

n/a	Confirmed	
☒	☐	The exact sample size (*n*) for each experimental group/condition, given as a discrete number and unit of measurement
☒	☐	A statement on whether measurements were taken from distinct samples or whether the same sample was measured repeatedly
☒	☐	The statistical test(s) used AND whether they are one- or two-sided<br>*Only common tests should be described solely by name; describe more complex techniques in the Methods section.*
☐	☒	A description of all covariates tested
☒	☐	A description of any assumptions or corrections, such as tests of normality and adjustment for multiple comparisons
☐	☒	A full description of the statistical parameters including central tendency (e.g. means) or other basic estimates (e.g. regression coefficient) AND variation (e.g. standard deviation) or associated estimates of uncertainty (e.g. confidence intervals)
☒	☐	For null hypothesis testing, the test statistic (e.g. *F*, *t*, *r*) with confidence intervals, effect sizes, degrees of freedom and *P* value noted<br>*Give P values as exact values whenever suitable.*
☒	☐	For Bayesian analysis, information on the choice of priors and Markov chain Monte Carlo settings
☒	☐	For hierarchical and complex designs, identification of the appropriate level for tests and full reporting of outcomes
☒	☐	Estimates of effect sizes (e.g. Cohen's *d*, Pearson's *r*), indicating how they were calculated

Our web collection on statistics for biologists contains articles on many of the points above.

Software and code

Policy information about availability of computer code

Data collection	The images were collected using NIKON NIS Elements.
Data analysis	Data was analyzed using Custom-built MATLAB codes developed by the group. The code is available on github (https://github.com/cecastro39/flochip/)

For manuscripts utilizing custom algorithms or software that are central to the research but not yet described in published literature, software must be made available to editors and reviewers. We strongly encourage code deposition in a community repository (e.g. GitHub). See the Nature Portfolio guidelines for submitting code & software for further information.

Data

Policy information about availability of data

All manuscripts must include a data availability statement. This statement should provide the following information, where applicable:

- Accession codes, unique identifiers, or web links for publicly available datasets
- A description of any restrictions on data availability
- For clinical datasets or third party data, please ensure that the statement adheres to our policy

The raw data pertaining all figures included in out manuscript are available on the open science framework (DOI 10.17605/OSF.IO/A6BFQ). The shared data includes time lapse files with Nikon NIS-Elements ND2 image format. Source data used to generate results presented in figures are provided with this paper.

Human research participants

Policy information about studies involving human research participants and Sex and Gender in Research.

Reporting on sex and gender	N/A
Population characteristics	N/A
Recruitment	N/A
Ethics oversight	N/A

Note that full information on the approval of the study protocol must also be provided in the manuscript.

Field-specific reporting

Please select the one below that is the best fit for your research. If you are not sure, read the appropriate sections before making your selection.

☒ Life sciences ☐ Behavioural & social sciences ☐ Ecological, evolutionary & environmental sciences

For a reference copy of the document with all sections, see nature.com/documents/nr-reporting-summary-flat.pdf

Life sciences study design

All studies must disclose on these points even when the disclosure is negative.

Sample size	Most force spectroscopy studies measure 10's-100's of interactions. For example (https://www.ncbi.nlm.nih.gov/pmc/articles/PMC1483847/). The high throughput nature of our system allows for much larger sample sizes greater than or comparable to other high throughput systems (https://www.nature.com/articles/ncomms11026).
Data exclusions	No data exclusion criteria was implemented. The beads that were non specifically interacting with the surface were detected and not included in the analysis.
Replication	All measurements were based on at least two different microfluidic devices prepared independently. Both attempts were successful.
Randomization	In all cases, to assess uncertainty, all force spectroscopy data was randomly divided into three different subgroups and analyzed based on replication without selection analysis methodology.
Blinding	No blinding strategy was implemented amongst the investigators. Blinding is not typically used in single molecule force spectroscopy.

Reporting for specific materials, systems and methods

We require information from authors about some types of materials, experimental systems and methods used in many studies. Here, indicate whether each material, system or method listed is relevant to your study. If you are not sure if a list item applies to your research, read the appropriate section before selecting a response.

Materials & experimental systems

n/a	Involved in the study	
☒	☐	Antibodies
☒	☐	Eukaryotic cell lines
☒	☐	Palaeontology and archaeology
☒	☐	Animals and other organisms
☒	☐	Clinical data
☒	☐	Dual use research of concern

Methods

n/a	Involved in the study	
☒	☐	ChIP-seq
☒	☐	Flow cytometry
☒	☐	MRI-based neuroimaging
